# Minor perturbations of thyroid homeostasis and major cardiovascular endpoints—Physiological mechanisms and clinical evidence

**DOI:** 10.3389/fcvm.2022.942971

**Published:** 2022-08-15

**Authors:** Patrick Müller, Melvin Khee-Shing Leow, Johannes W. Dietrich

**Affiliations:** ^1^Department for Electrophysiology, Medical Hospital I, Klinikum Vest, Recklinghausen, NRW, Germany; ^2^Singapore Institute for Clinical Sciences (SICS), Agency for Science, Technology and Research (A^*^STAR), Singapore, Singapore; ^3^Department of Endocrinology, Tan Tock Seng Hospital, Singapore, Singapore; ^4^Metabolic Disorders Research Programme, Lee Kong Chian School of Medicine, Singapore, Singapore; ^5^Cardiovascular and Metabolic Disorders Program, Duke-NUS Medical School, Singapore, Singapore; ^6^Diabetes, Endocrinology and Metabolism Section, Department of Internal Medicine I, St. Josef Hospital, Ruhr University Bochum, Bochum, NRW, Germany; ^7^Diabetes Centre Bochum/Hattingen, St. Elisabeth-Hospital Blankenstein, Hattingen, NRW, Germany; ^8^Centre for Rare Endocrine Diseases, Ruhr Centre for Rare Diseases (CeSER), Ruhr University Bochum and Witten/Herdecke University, Bochum, NRW, Germany; ^9^Centre for Diabetes Technology, Catholic Hospitals Bochum, Ruhr University Bochum, Bochum, NRW, Germany

**Keywords:** thyroid function, sudden cardiac death, ventricular arrhythmia, cardiac electrophysiology, MACE, hypothyroidism, thyrotoxicosis, type 2 allostatic load

## Abstract

It is well established that thyroid dysfunction is linked to an increased risk of cardiovascular morbidity and mortality. The pleiotropic action of thyroid hormones strongly impacts the cardiovascular system and affects both the generation of the normal heart rhythm and arrhythmia. A meta-analysis of published evidence suggests a positive association of FT4 concentration with major adverse cardiovascular end points (MACE), but this association only partially extends to TSH. The risk for cardiovascular death is increased in both subclinical hypothyroidism and subclinical thyrotoxicosis. Several published studies found associations of TSH and FT4 concentrations, respectively, with major cardiovascular endpoints. Both reduced and elevated TSH concentrations predict the cardiovascular risk, and this association extends to TSH gradients within the reference range. Likewise, increased FT4 concentrations, but high-normal FT4 within its reference range as well, herald a poor outcome. These observations translate to a monotonic and sensitive effect of FT4 and a U-shaped relationship between TSH and cardiovascular risk. Up to now, the pathophysiological mechanism of this complex pattern of association is poorly understood. Integrating the available evidence suggests a dual etiology of elevated FT4 concentration, comprising both ensuing primary hypothyroidism and a raised set point of thyroid function, e. g. in the context of psychiatric disease, chronic stress and type 2 allostatic load. Addressing the association between thyroid homeostasis and cardiovascular diseases from a systems perspective could pave the way to new directions of research and a more personalized approach to the treatment of patients with cardiovascular risk.

## Introduction

Sudden cardiac death (SCD) is a global health issue that causes more than 600,000 fatalities per annum in the United States and Europe alone (1–3) being responsible for 15–20% of total mortality in industrialized societies (4–7). Therefore, the prevention of SCD continues to be a major task of cardiovascular medicine (8). Several conditions are known to be associated with increased risk of SCD including higher age, male sex, coronary artery disease (previous myocardial infarction), cardiomyopathies, primary electrical disorders, aortopathies or aortic dissection, and decreased left ventricular systolic function (7, 9). The main underlying pathologic substrate that conveys the development of ventricular tachyarrhythmias responsible for SCD involves channelopathies and/or the presence of myocardial fibrosis.

With the advent of implantable cardioverter-defibrillators (ICDs), accurate assessment of risk for SCD becomes crucial in clinical practice, the more as the majority of all first clinical events is fatal (10). Current guidelines on primary prevention for SCD recommend risk stratification solely on heart failure symptoms and reduced left ventricular ejection fraction (LVEF) (11–14). Several studies confirmed LVEF as a strong predictor of arrhythmic death (15, 16). For example, the Autonomic Tone and Reflexes After Myocardial Infarction (ATRAMI) study revealed that LVEF <35% was associated with a relative risk for cardiac mortality of 7.3 in short-term survivors of myocardial infarction (17, 18). Moreover, randomized controlled ICD trials for primary prevention in patients with reduced LVEF demonstrated improved survival within the ICD arms (19). However, using LVEF as a sole risk stratifier for SCD has several limitations. First, a relevant number of SCD continues to occur in subjects with LVEF > 40%. The Maastricht Circulatory Arrest Registry reported that more than 50% of victims of sudden circulatory arrest had LVEF > 40% (20, 21). Therefore, a significant proportion of those at risk for SCD live unnoticed when stratified by LVEF alone. Second, even in patients with LVEF <35% only a small proportion will benefit from ICD. Recent data of the DANISH trial revealed a low incidence of appropriate ICD shocks (11.5%) in patients with non-ischemic cardiomyopathy after a median follow-up of 67.6 months, whereas on the other hand the incidence of device infection (4.9%) and inappropriate shocks (5.9%) was considerable (22). Third, further developments of heart failure medications such as the angiotensin-neprilysin inhibitor combination LCZ696 (sacubitril/valsartan) may improve LVEF and minimize the risk for SCD (23–25).

Therefore, it is highly needed to identify additional markers potentially predicting a higher relative risk for SCD (26). This form of biomarkers would also open avenues to a better understanding, which are the patients that mainly benefit from ICD treatment (27).

Among prevalent, but previously underrecognized conditions, hyperthyroidism and thyrotoxicosis are known to significantly increase the risk for cardiac morbidity and mortality (28–33). Elevated concentration of free thyroxine (FT4) in overt thyrotoxicosis is an established risk factor for major cardiovascular endpoints (including cardiovascular death, hospital admission, ventricular arrhythmia and ICD therapy) (28, 30, 31). Subclinical thyroid disorders and “euthyroid” variations of FT4 within their respective reference range have been linked to cardiovascular outcome measures as well, but their mechanisms are less well understood (34, 35). Likewise, the implications of altered thyrotropin (TSH) concentration continue to be unclear (36–39). Although major cardiovascular endpoints define an important health issue, the available evidence from the literature is ambiguous, leaving important questions unsolved (29, 37).

Despite the established pro-arrhythmic role of thyroid hormones, treatment with levothyroxine may have favorable effects in heart failure (40), and both subclinical and overt hypothyroidism are established risk factors for coronary heart disease and mortality (41). The line between beneficial and harmful effects of substitution therapy, is small, however, since thyrotoxicosis and even subclinical hyperthyroidism may be independent triggers for heart failure and cardiovascular mortality (42–44). In two large population-based studies even the use of antithyroid drugs was found to be a risk factor for SCD (45), but causality remains unclear.

Until recently, the question could not be resolved if screening of subclinical and undiagnosed overt thyroid dysfunction and treatment of subclinical hypothyroidism is beneficial in adults without goiter or thyroid nodules (46). Potential reasons for this persisting vagueness include the complexity of thyroid hormone action in the cardiovascular system, age dependence of the involved effects and misconceptions about the role of thyrotropin in thyroid homeostasis, as well as insufficient sample size and heterogeneity of study designs (37, 38, 47–52).

Our systematic analysis was motivated by the hypothesis that both TSH and T4 concentrations are associated to important end points of cardiac arrhythmia, but that the specific relationships are different from a qualitative perspective. The underlying differences might arise from the dual role of both TSH and thyroid hormones, acting as controlling as well as controlled elements, from the inverse relationship between T4 and TSH in primary thyroid dysfunction and from the fact that TSH (and TRH) secretion is controlled by multiple central afferences reflecting the role of the TRH neuron as an integrator of stress signaling and energy homeostasis (53–59).

This review article is organized in four parts. In the next section we summarize known fundamental concepts including historical notes and insights from basic research. The subsequent section covers the methodology and results of a systematic search and meta-analysis of the association of mild disorders of thyroid homeostasis with major adverse cardiovascular endpoints (MACE). Toward the end of the article, we discuss the implications of the findings and try to integrate the available evidence in a comprehensive model that provides a possible explanation of all observations including previously poorly understood phenomena as well.

## Fundamental physiological evidence

Thyroid hormones are key regulators of growth, differentiation and integrative energy homeostasis. They orchestrate the trade-off between–often conflicting–demands of supply with energy and substrates, ontogeny, thermoregulation and fight-and-flight reactions (56). As slow mediators of allostatic load, they represent the fourth level of the stress response after the sensorimotor and autonomic nervous system, the release of catecholamines and the secretion of glucocorticoids (56). It is therefore not surprising that their role as a switch between anabolic and catabolic functions includes the cardiovascular system as well. Cardiac complications of thyrotoxicosis were probably first observed as early as 1,785 by Caleb Hillier Parry, who portrayed a series of cases with exophthalmic goiter that was associated with palpitations and tachycardia (60), shortly later followed by similar observations made in other countries (61–63).

Today it is well recognized that a broad spectrum of heart diseases may ensue from disorders of thyroid homeostasis (31, 32, 64). Established scoring systems for the diagnosis of myxoedema coma and thyroid storm, two life-threatening thyroid conditions, comprise cardiac manifestations including brady- and tachycardia, congestive heart failure and atrial fibrillation (30).

**Figure 1:**
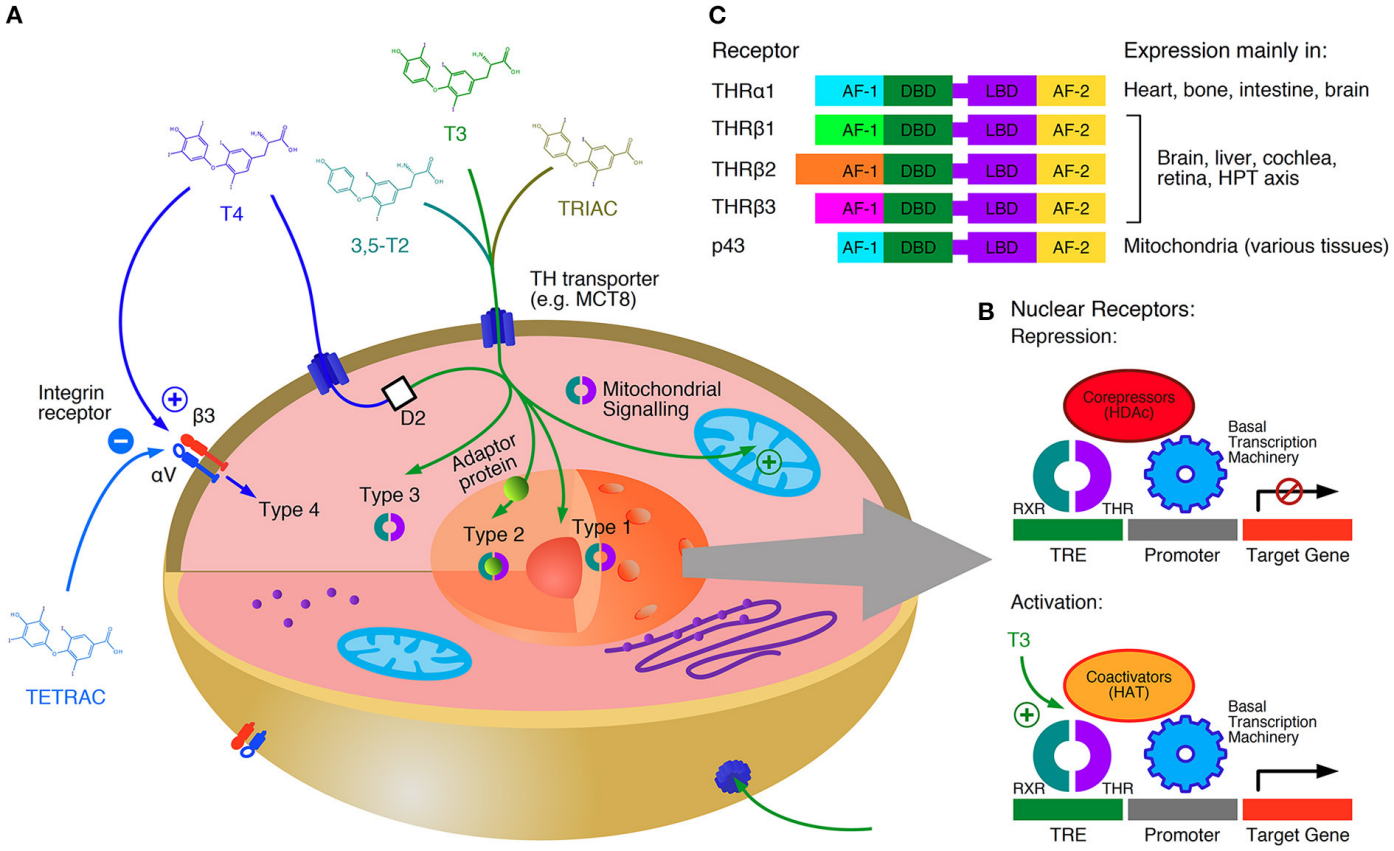
Types and mechanisms of thyroid hormone signaling. T4 is a prohormone with respect to genomic signaling, but a true fast-acting hormone regarding type 4 action (which is inhibited by the iodothyroacetate TETRAC). Genomic action (type 1, type 2 and mitochondrial signaling) occurs on a slow time scale, whereas non-genomic effects (type 3 and type 4 signaling) represent a fast response **(A)**. Thyroid hormone receptors (THR) usually act as heterodimers. Without thyroid hormone bound they block the transcription together with corepressors. Iodothyronines displace the corepressors and stimulate gene expression together with coactivators **(B)**. Tissue specific distributions of THRs further contribute to the diversity of signaling patterns in the organism **(C)** (48, 65–69). AF-1, activation function 1; AF-2, activation function 2; D2, type 2 deiodinase; DBD, DNA-binding domain; HAT, histone acetyl-transferase; HDAc, histone deacetylase; LBD, ligand-binding domain; RXR, retinoid X receptor; TRE, thyroid-hormone response element.

On a molecular and cellular level pleiotropic effects of iodothyronines on gene expression, metabolism and electrophysiological mechanisms have been described (64). The effects of thyroid hormones are mediated by four different signaling types (Figure 1) (48). Type 1 of thyroid hormone action represents classical, genomic effects. Here, iodothyronines dock to thyroid hormone receptors (THR) bound to DNA as monomer, homodimer or heterodimer and modify the expression of regulated genes. THRs may also bind to RNA *via* a specific binding site, such that their binding and recruitment of RNA can accentuate its transcriptional activity of thyroid hormone responsive genes (70). Five types of THRs are able to both bind thyroid hormones and form active dimers, THRα1, which is the main receptor in myocardial tissue and bone, THRβ1, THRβ2 and THRβ3, which play an important role in extracardiac tissue (e.g., pituitary, liver and adipose tissue) (65) and the mitochondrial p43 receptor (71). In type 2 signaling, hormone-receptor complexes are indirectly bound to DNA *via* adaptor proteins. In type 3 action, iodothyronines are bound to (cytoplasmic) thyroid hormone receptors, and their effects are mediated *via* intracellular transmitters (second messengers), e. g. the MAPK and PI3K/AKT/mTOR pathways. The term Type 4 signaling refers to responses mediated *via* integrin receptors on the cell membrane (48). Of note, genomic effects (type 1 and 2 signaling) occur on a much slower time scale (hours) than non-genomic effects (type 3 and 4 signaling, minutes) (66, 72). The signaling types 1 to 3 require two iodine atoms at the inner ring of the hormone molecule, but a maximum of one iodine atom bound to the outer ring. Therefore, triiodothyronine (T3), triiodothyroacetic acid (TRIAC) and 3,5-diiodothyronine (3,5-T2) exert strong and direct genomic and non-genomic effects, whereas thyroxine (T4) and tetraiodothyroacetic acid (TETRAC) act directly *via* non-genomic signaling only, and only indirectly (after deiodination to T3, 3,5-T2 or TRIAC) *via* genomic signaling (67, 73, 74). It could be recently demonstrated that the effects of thyroid hormones on the heart rate involve type 3 signaling in a complex interaction with the autonomic nervous system (66).

The transcription of multiple genes is stimulated by classical thyroid hormone signaling. They include alpha-myosin heavy chain, atrial natriuretic hormone, beta1- and beta-2-adrenergic receptors, sarcoplasmic reticulum Ca^2+^-ATPase (SERCA), Na^+^/K^+^-ATPase, and voltage-gated potassium channels (Kv1.5, Kv4.2, Kv4.3). Negatively regulated genes include adenylyl cyclase catalytic subunits, beta-myosin heavy chain, phospholamban, THRα1 and the Na^+^/Ca^2+^ exchanger. Animal experiments revealed complex remodeling of cardiac ion channel expression depending on thyroid status (75). Fast responses of ion channels result from direct modulation *via* non-genomic type 4 signaling (31, 64, 76).

Additional effects on cardiovascular physiology may result from extracardiac mechanisms in the cardiovascular system, e. g., increased pulmonary arterial pressure, stimulation of tissue thermogenesis and a decline of both systemic vascular resistance and diastolic blood pressure, resulting in reduced afterload (31, 49, 64, 76–79).

### Links between thyroid hormone action and arrhythmogenesis

The mechanisms underlying arrhythmogenesis can be divided into disorders of impulse formation, disorders of impulse conduction or a combination of both (80). All of these scenarios can be critically modulated by thyroid hormones (Table 1).

**Table 1:** Mechanisms of arrhythmia in different thyroid conditions (76, 81–84).

**Disorder**	**Hypothyroidism**	**Thyrotoxicosis**
**Disorders of impulse formation**		
**Automaticity**		
Normal automaticity	Sinus bradycardia	Sinus tachycardia
**Triggered activity**		
Early afterdepolarisations		Ventricular tachyarrhythmia
Delayed afterdepolarisations		Atrial and ventricular sustained triggered arrhythmia
**Disorders of impulse conduction**		
**Block and re-entry**		
Uni- or bidirectional block without re-entry	SA block AV block	Decremental conduction with paradox bradycardia or heart block
Unidirectional block with		Atrial fibrillation
re-entry		Ventricular flutter
		Ventricular fibrillation
		Ventricular tachycardia

#### Effects of thyroid hormones on normal automaticity

Within the framework of their normal physiological function thyroid hormones have strong effects on the heart rate. Sinus bradycardia and sinus tachycardia are typical sequelae of hypothyroidism and thyrotoxicosis, respectively.

**Figure 2:**
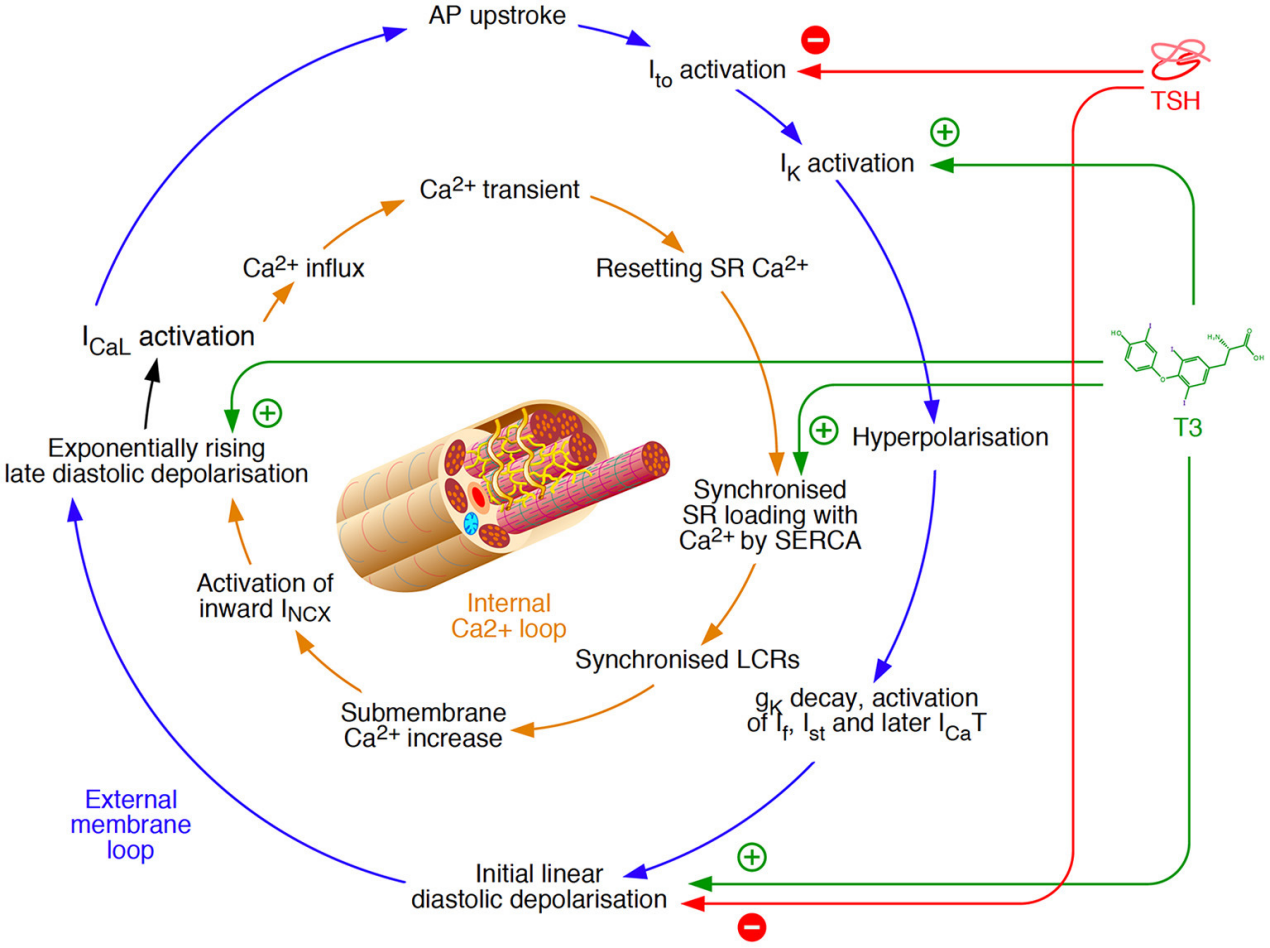
Mechanisms of rhythm generation in cardiomyocytes based on a model by Maltsev et al. (85). Two loops, an external membrane loop and an internal calcium loop independently ensure the generation of impulses. Therefore, they provide some redundancy, but they are also intertwined at the stage of slow L-type Ca^2+^ current (I_CaL_) activation. Thyroid hormone signaling is able to modulate both loops simultaneously *via* interfaces at several sites (75, 81, 86–91), and direct myocardial effects of TSH are largely opposing (92). g_K_, ionic conductance for K^+^; INCX, Na^+^/Ca^2+^ exchange current; I_CaT_, T-type Ca2+ current; I_f_, hyperpolarisation-activated “funny” current; I_K_, voltage-gated K^+^ current; I_st_, sustained non-selective current; I_to_, transient outward potassium current; LCR, local Ca^2+^ release; SERCA, sarcoendoplasmic reticulum Ca^2+^-ATPase; SR, sarcoplasmic reticulum.

This association is well explained by modulation of the impulse formation in pacemaker cells of the sinoatrial node and (in pathological conditions) other cell types. The normal automaticity in pacemaker cells is ensured by two redundant, but intertwined loops, as suggested by Maltsev et al. (85) (Figure 2). Both an external membrane loop and an internal calcium loop, buffered by calcium storage in the sarcoplasmic reticulum, are independently able to maintain the generation of a depolarisation rhythm. They are coupled *via* late diastolic depolarisation and a slow L-type calcium current, thereby providing a better failure tolerance in impulse generation. Thyroid hormones can modulate both to generate a higher frequency of action potentials. The involved mechanisms include an acceleration of diastolic depolarisation (86–88), activation of potassium currents (81, 89) and synchronized loading of the sarcoplasmic reticulum with calcium *via* SERCA (75, 90, 91).

In addition to thyroid hormones, thyroid-stimulating hormone (TSH) is able to modulate the function of the sinoatrial node as well. Among its extrathyroidal actions are myocardial effects that are mediated *via* G protein-coupled TSH receptors (TSHR), which are expressed in various tissues. The resulting impact of TSH on the physiological rhythm generator is mainly of decelerating nature by inhibiting several ion currents (Figure 2) (92). Since the effects of TSH action are largely antagonistic to those of beta-adrenergic stimulation they are probably mediated by pathways other than the cAMP signaling, e.g., *via* inositol trisphosphate (IP3) (93, 94).

Although we write about the “normal heart rhythm” and “physiological function” here, the mentioned mechanisms may lead to substantial pathology, e. g., in cases of severe bradycardia or tachycardia in thyroid dysfunction or activation of ectopic rhythm generators. “Normality” refers to the fact that it is physiological mechanisms that are controlled by thyroid hormone signaling. This is different in the situations mentioned in the subsequent section, which ensue from a qualitative difference and represent pathological processes *per se*.

#### Mechanisms linking thyroid dysfunction to cardiac arrhythmia

In addition to the modulation of physiological mechanisms, high concentrations of thyroid hormones can ignite triggers for additional scenarios of arrhythmogenesis.

Afterdepolarisations, i.e., depolarising oscillations of membrane potential after one or more preceding action potentials, are able to initiate myocardial activity. Both early (occurring before full repolarisation) and delayed afterdepolarisations (arising after completion of repolarisation) can be mediated by elevated concentrations of thyroid hormones, giving rise to triggered activity (81).

**Figure 3:**
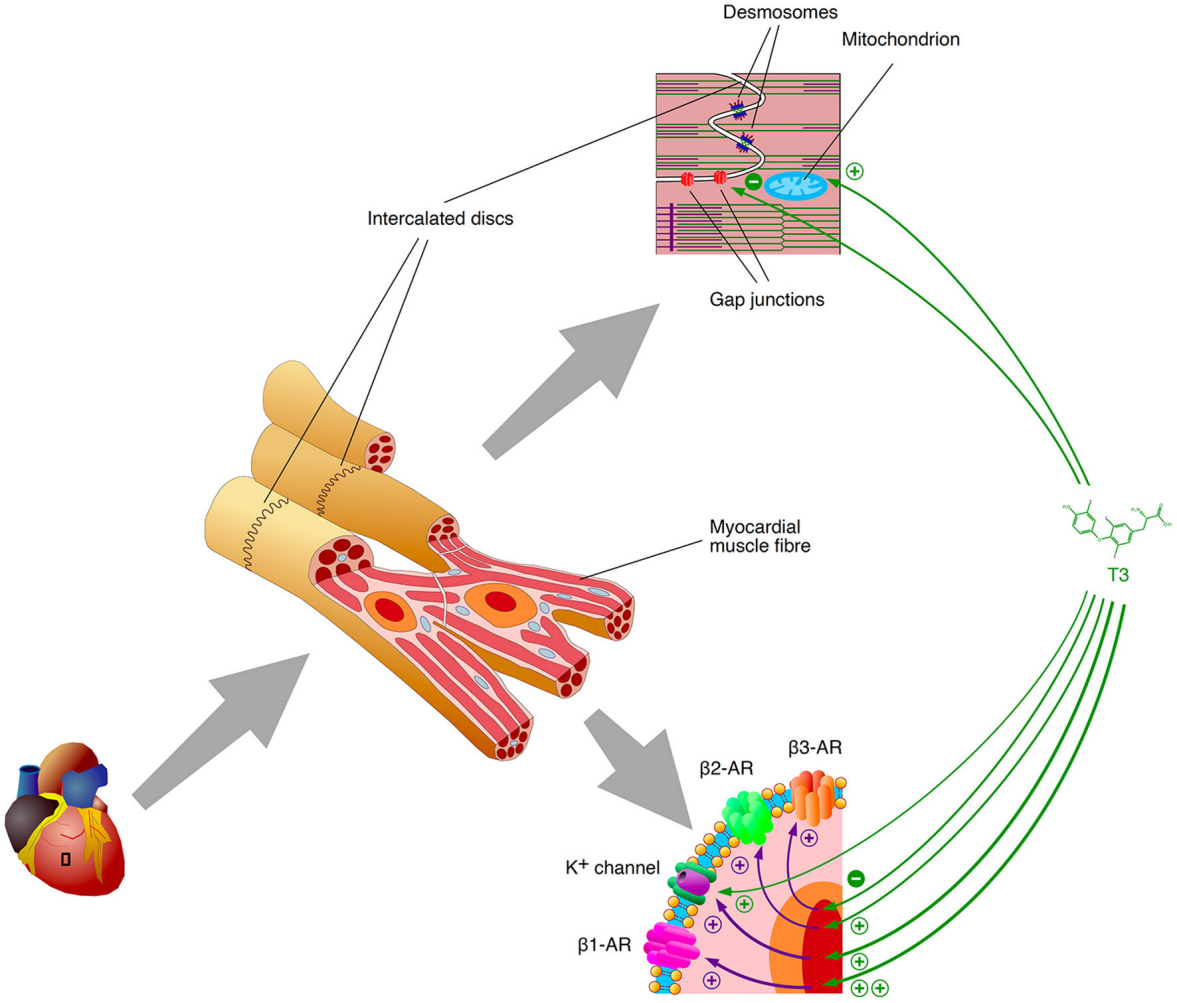
Selected mechanisms of arrhythmogenesis by thyroid hormones. T3 (and other active thyroid hormones) upregulate the gene expression of beta1 and beta2 adrenoceptors *via* classical genomic signaling, but downregulate protective beta3 adrenoceptor expression. The transcription of critical genes for the formation of gap junctions is downregulated as well. Potassium channels are regulated *via* classical type 1 signaling and *via* non-genomic effects (type 4 action) as well. Purple arrows indicate the effects of gene expression (transcription, translation and associated processing steps), green arrows visualize the impact of T3-agonistic thyroid hormones.

**Figure 4:**
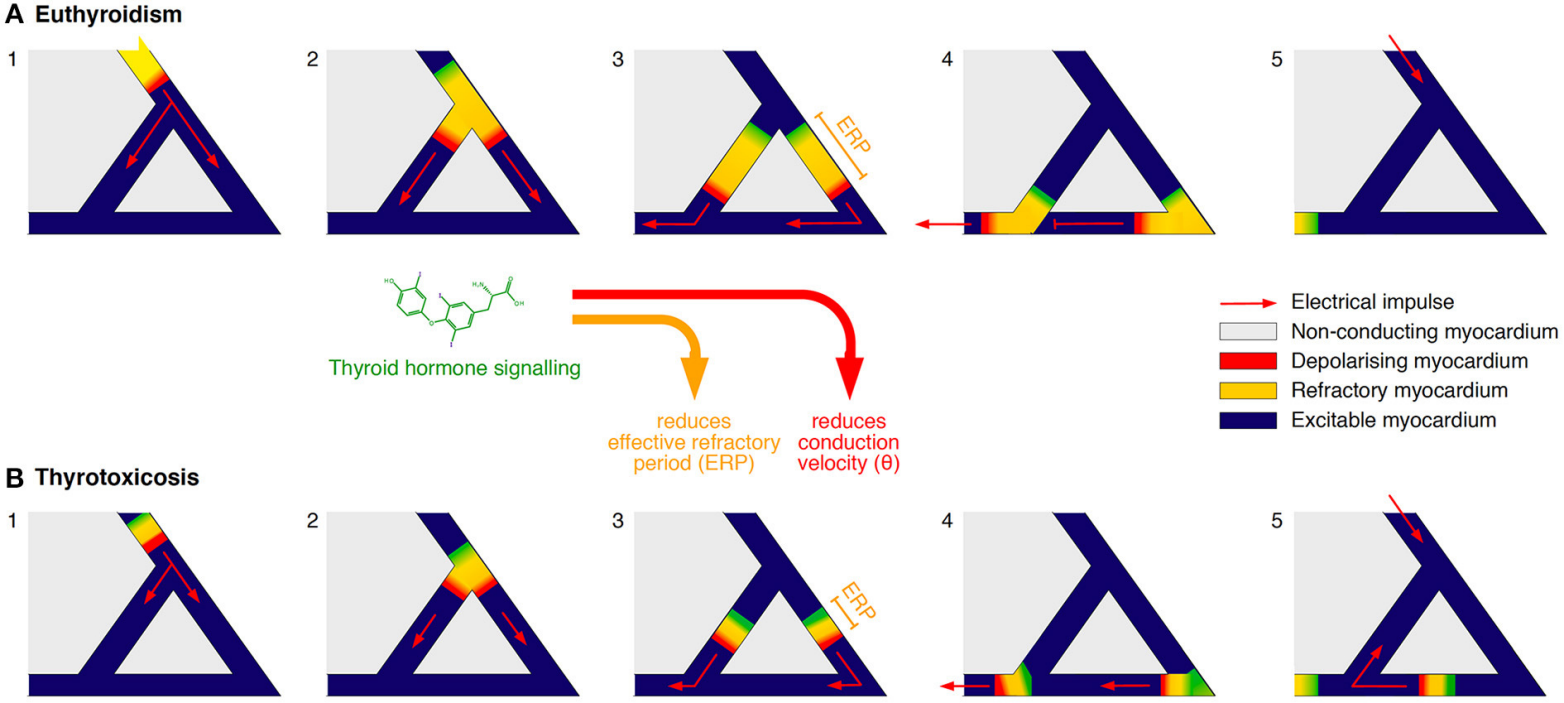
**(A,B)** Simplified model of the effects of thyroid hormone signaling facilitating disorders of impulse conduction. Iodothyronine action reduces both the effective refractory period (*ERP*) and the conduction velocity (θ). As a consequence, the wavelength of excitation λ = *ERP* x θ may get shorter than the dimensions of a potential re-entry circuit, thus giving rise to re-entrant tachycardia (99).

Additionally, iodothyronines shorten the effective refractory period (ERP) of cardiomyocytes in a concentration-dependent manner (95–97), and they reduce the conduction velocity (θ), e.g. by downregulating the expression of connexin 43, a key component of gap junctions (Figure 3) (75, 96–98). As a result, the wavelength of excitation (λ), which is defined by the product of *ERP* and θ, is considerably diminished, so that it may become shorter than the dimensions of potential re-entry circuits, e.g., in the vicinity of scars (99) (Figure 4). Consequently, an electrical impulse traversing a cardiac fiber bundle is slowed down and may, provided electrical irregularities are present in the tissue, meet excitable fibers after the refractory period has passed. Therefore, the combination of both mechanisms permits the evolution of re-entry or spiral wave processes (100).

The probability for successful action potential propagation depends on the safety factor for conduction, i.e., the ratio of the energy for normal conduction to the minimum propagating energy (80). Impaired function of gap junctions may lead to decremental conduction, where an impulse with a low safety factor loses activation effectiveness along with its propagation. This mechanism may cause rare paradoxical bradycardia or heart blocks in thyrotoxicosis (82, 83).

### Controversial topics and open questions

The term of “subclinical” thyroid diseases continues to be debated. It is unclear if this class of diseases is a proper representation of physiological processes (101–104), and the implications with respect to meaningful endpoints are unclear (105, 106). Therefore, useful cut-off values serving as a threshold for initiating therapy, could not be established (35). Recently, a more detailed grading system has been suggested, including FT3 and different zones of TSH concentration (107). It may be beneficial for a better classification of primary thyroid dysfunction.

This new classification cannot, however, address the dual role of TSH as both a regulator and indicator of thyroid function (53, 102, 103, 108, 109). Deviations of thyrotropin concentration may indicate ensuing or manifest primary thyroid disorders, but pituitary disease and variations in the central hypothalamic set point as well (37, 47). This physiological heterogeneity may explain why thyroid hormones are better predictors of clinical outcome measures than TSH concentration (38).

Furthermore, universal reference ranges for thyrotropin and thyroid hormones have recently been more and more questioned, since the intraindividual variation of TSH and thyroid hormone concentration is smaller than the interindividual variation (110, 111). This non-ergodicity of laboratory results reflects an individual set point of the homeostatic systems, and deviations from this setpoint may from a personalized perspective be of higher importance than the position of laboratory results with respect to population-derived reference ranges (37, 112–116).

## Clinical evidence

### Review criteria

To compile the most comprehensive list of pertinent publications electronic literature searches in English, German and Mandarin were performed in the PubMed and Web of Science databases for relevant publications up to December 2021, evaluating a potential connection between minimal disorders of thyroid function and sudden cardiac death or malignant arrhythmia. The following query formula was used: “[(thyroid function) OR (free t4)] AND [(sudden cardiac death) OR (ventricular fibrillation) OR (ventricular tachycardia)]”. Our search was limited to studies that were performed in humans and investigated the thyroid function in relation to sudden cardiac death, a combination of major endpoints including cardiovascular death (CVD) or the result of continuous recording *via* an implanted device (pacemaker, ICD or event recorder).

Among the larger set of identified studies, the meta-analysis subproject was restricted to longitudinal investigations that reported quantitative information on thyroid homeostasis and a hazard or odds ratio for one of the mentioned cardiovascular endpoints. If adjusted and unadjusted analyses with the same outcome measures and thyroid-related predictors were reported in one paper a fully adjusted multivariable analysis was selected for further evaluation.

Exclusion criteria were case reports, animal or cell culture experiments, therapeutic trials, studies on non-thyroidal illness (TACITUS) syndrome, studies on amiodarone effects, surveys, review articles or correspondence without original data. After removing duplicates, three authors (P.M, M.K.L and J.W.D.) screened all found studies for eligibility by abstract screening and full-text reviewing. From the included publications quantitative data, including sample size, mean and standard error (or 95% confidence interval) of hormone concentrations, hazard ratio or odds ratio for cardiovascular endpoints and type of study (prospective or retrospective) were extracted.

In order to address a potentially low number of studies and to reduce the effects of bias arising from heterogeneities in MACE definitions (117–119), we used a hierarchical approach, where we analyzed the association of thyroid function separately with SCD, CVD, study-specific MACE (as defined by the authors of included publications) and inclusive MACE (the union set of MACE and cardiovascular death).

For studies reporting FT4 concentration in other units of measurement than pmol/L, HRs were converted with


$$\begin{array}{l} HR_{C}=1+\left(HR-1\right)\cdot conversion\;factor. \end{array}$$

Likewise, if TSH concentration was reported in logarithmic presentation, the HR was adjusted with


$$\begin{array}{l} HR_{C}=1+\frac{HR-1}{e^{1}} \end{array}$$

In studies that reported HRs for increases of TSH and FT4 in standard deviations adjustments were performed with


$$\begin{array}{l} HR_{C}=1+\frac{HR-1}{SD} \end{array}$$

The quality of included studies was assessed with the Newcastle-Ottawa score (NOS). In order to control for small-study effects leading to potential bias, funnel plots were drawn for analyses with five or more studies. Quantitative papers were pooled and summary measures were included in random-effects meta-analysis. The between-study variance was assessed with the DerSimonian-Laird estimator, Cochran's Q and tau squared, and heterogeneity with Higgins' and Thompson's *I*^2^. Calculations were performed with custom S scripts for the statistical environment R (version 4.1.1 for macOS) and supported by the R packages meta and metaphor.

The protocol for the meta-analysis has been registered in the PROSPERO International prospective register of systematic reviews with the ID CRD42022311340 and is available from https://www.crd.york.ac.uk/prospero/display_record.php?ID=CRD42022311340.

### Results of longitudinal studies

**Figure 5:**
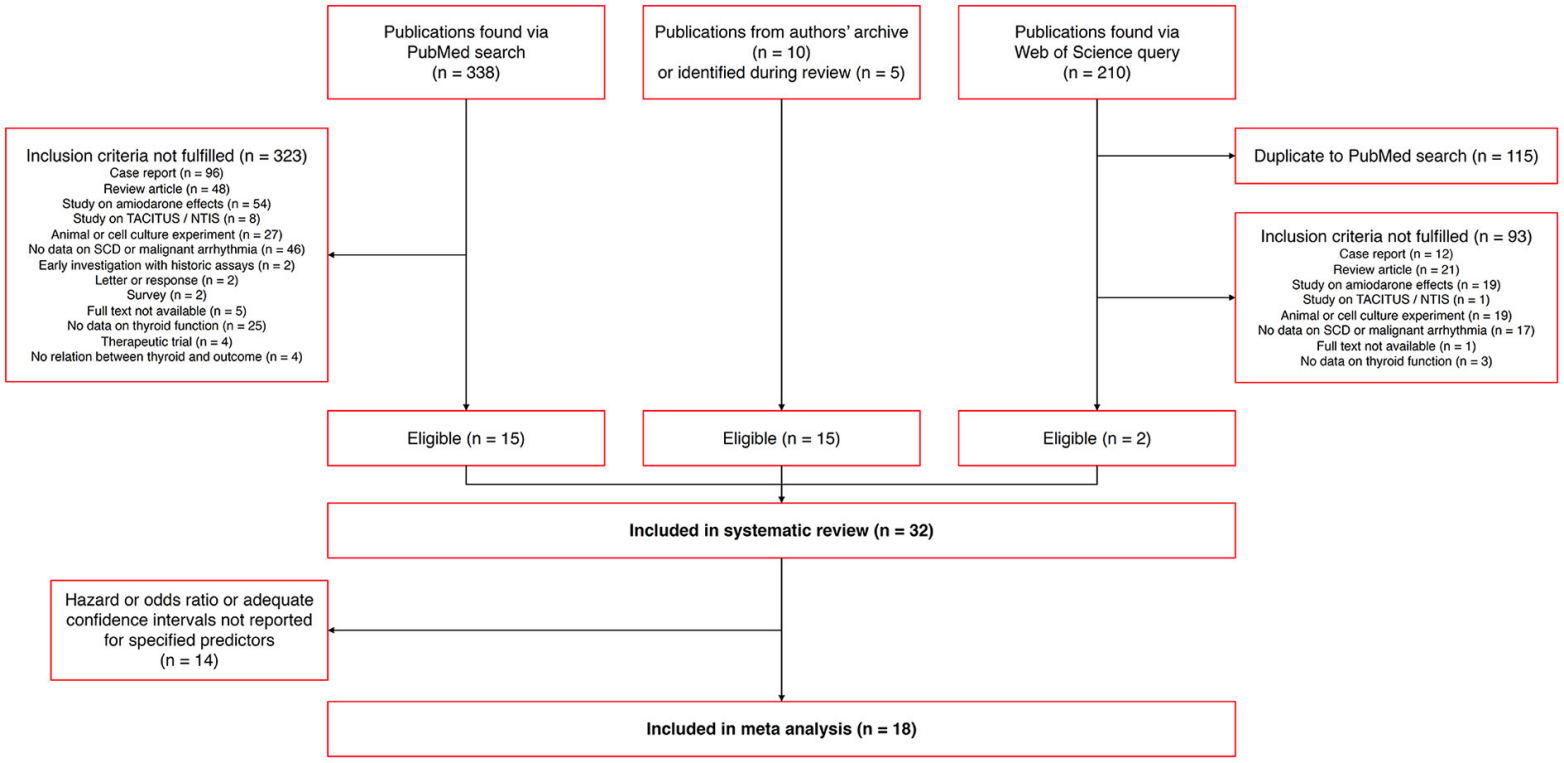
Flowchart of identified, eligible and included publications.

*Via* the described search strategy and other sources, we identified 32 eligible publications that were included in the systematic review (Figure 5). From these, 18 studies reported an association of thyroid homeostasis to the hazard ratio of SCD, CVD, ICD therapy or major cardiovascular events comprising one of these outcome measures. These studies could be included in the meta-analysis. See Supplementary figure 1 for the numbers of included studies with respect to different outcome measures.

Seven studies, including more than 1.2 million subjects, observed an association of reduced TSH concentration with major cardiovascular endpoints (Table 2). A very large register-based study including more than half a million citizens of Copenhagen found reduced TSH concentration to be associated with MACE, heart failure and all-cause mortality (42). In another large cohort study with elderly community-dwelling individuals diminished TSH levels were associated with a higher incidence of all-cause death (121). In a similar study with subjects aged 85 years or older reduced TSH concentration predicted both all-cause and cardiovascular mortality (120). A prospective multicentre cohort study observed a suppressed TSH concentration to be associated with higher cardiovascular mortality and risk of stroke. However, after multivariable association it was associated with stroke only (123). Another multicentre cohort study observed increased all-cause mortality in subjects with systolic heart failure and reduced TSH concentration, but the association vanished in an adjusted model (124). A study with 1 000 diabetic patients on haemodialysis found a reduced TSH level to be associated with a doubled risk of SCD, and this effect was preserved in an adjusted model (126).

**Table 2:** Association between TSH concentration and major cardiovascular outcome measures.

**Study**	**Study population**	**Number of included subjects**	**Study design**	**Evaluation period**	**Outcome**	**Main results**
Gussekloo et al. (120)	Population-based sample of elderly subjects aged 85 years or older	599	Population-based prospective cohort study	Mean 3.7 years	All-cause mortality, disability, depressive symptoms, cognitive function	Reduced TSH concentration was associated with higher all-cause and cardiovascular mortality rate.
Cappola et al. (121)	Community-dwelling individuals aged 65 years or older	3,233	Population-based prospective cohort study	Mean 12.5 years	All-cause mortality, coronary heart disease, cerebrovascular disease, atrial fibrillation	Reduced TSH was associated with higher incidence of all-cause death and atrial fibrillation.
Razvi et al. (122)	Community-dwelling subjects from the Wickham survey	2,376	Population-based prospective cohort study	Up to 20 years	Incidence and mortality of ischaemic heart disease (IHD)	Elevated TSH concentration in subclinical hypothyroidism associated with higher rate of fatal and nonfatal events and mortality of IHD.
Schultz et al. (123)	Random sample from general practitioners aged 50–91 years with normal LVEF	605	Prospective multicentre cohort study	Median 5 years	All-cause and cardiovascular mortality, stroke	Reduced TSH was associated with higher cardiovascular mortality and risk of stroke (after multivariable adjustment associated with stroke only)
Frey et al. (124)	Subjects with systolic heart failure	758	Prospective multicentre cohort study	3 years	All-cause mortality	Reduced TSH was associated with increased mortality in unadjusted model. No association in adjusted model.
Mitchell et al. (125)	Subjects with heart failure	2,225	Prospective multicentre cohort study	Median 3.8 years	All-cause mortality	Both reduced and elevated TSH was associated with increased mortality.
Drechsler et al. (126)	Diabetic haemodialysis patients	1,000	Prospective multicentre cohort study	4 years	All-cause mortality, sudden cardiac death, stroke, combined CV events (sudden death, MI or stroke)	Reduced TSH was associated with doubled risk of sudden cardiac death (in adjusted and unadjusted models). Elevated TSH was not associated with outcome measures.
Perez et al. (127)	Patients with systolic heart failure	4,987	Prospective multicentre cohort study	Median 2.7 years	Cardiovascular mortality, nonfatal myocardial infarction, nonfatal stroke	Elevated TSH was associated with increased all-cause and cardiac mortality in unadjusted model. No association after adjustment.
Selmer et al. (42)	Citizens of Copenhagen without previous thyroid dysfunction undergoing thyroid function testing	563,700	Population-based prospective cohort study	Median 5.5 years	Myocardial infarction (MI), heart failure, stroke, composite MACE (CVD, nonfatal MI or nonfatal stroke) and all-cause mortality	Reduced TSH concentration was associated with MACE, heart failure and all-cause mortality. Elevated TSH was associated with MI.
Chaker et al. (36)	Community-dwelling individuals included in the Rotterdam study	10,318	Population-based prospective cohort study	Median 9.1 years	Sudden cardiac death	TSH concentration was not associated with risk for sudden cardiac death
Rhee et al. (128)	Patients receiving peritoneal dialysis	1,484	Prospective multicentre cohort study	Median 1.0 years	All-cause mortality.	Both reduced and elevated TSH was associated with increased mortality (unadjusted and adjusted models).
Pearce et al. (129)	Members of the Newcastle 85+ study, recruited from general (family) practices	643	Prospective cohort study	Up to 9 years	All-cause and cardiovascular mortality	TSH concentration was not associated with all-cause or cardiovascular mortality after adjustment.
Langén et al. (130)	Community-dwelling individuals aged ≥ 30 years	5,211	Population-based prospective cohort study	Median 13.2 years	All-cause mortality, sudden cardiac death, CHD events, CVD, stroke, MACE (CVD or heart failure), AF	Elevated TSH was associated with increased all-cause mortality and SCD, no association to other outcomes. U-shaped association of TSH to total mortality after spline transformation.
Kannan et al. (131)	Patients with heart failure enrolled in the Penn Heart Failure Study	1,365	Prospective multicentre cohort study	Median 4.2 years	Composite end point of all-cause mortality, cardiac transplant or VAD placement.	Elevated TSH was associated with increased hazard for composite end point.
Golledge et al. (132)	Community-recruited elderly men without known thyroid disease	3,712	Population-based prospective cohort study	Mean 9.5 years	Composite end point of cardiovascular death, myocardial infarction or stroke	TSH concentration or its quartiles were not associated to the composite end point.
Li et al. (133)	Euthyroid patients with nonischemic dilated cardiomyopathy	184	Prospective unicentre cohort study	Median 4.6 years	All-cause and cardiac mortality, events of ventricular arrhythmia, exacerbation of heart failure, heart transplant	TSH concentration within its reference range was positively associated with risk for VA events (unadjusted and adjusted models). No association to other outcome measures.
Seo et al. (134)	Patients with acute myocardial infarction	1,977	Prospective multicentre cohort study	Median 3.5 years	All-cause and cardiac mortality	Elevated TSH was associated with higher all-cause and cardiac mortality.
Groothof et al. (39)	Community-dwelling euthyroid individuals aged 28–75 years	6,054	Population-based prospective cohort study	Mean 7.9 years	All-cause and cardiovascular mortality	In subjects younger than 72 years TSH concentration within reference range was positively associated with cardiovascular mortality in adjusted model.
Inoue et al. (135)	Community-dwelling individuals included in the NHANES study	9,020	Population-based prospective cohort study	Median 7.3 years	All-cause mortality	Low-normal, high-normal and elevated TSH was associated with higher all-cause mortality than middle-normal TSH, partly mediated by cardiovascular disease.
Kim et al. (136)	Patients undergoing cardiac surgery	565	Retrospective unicentre case-control study	Mean 7.6 years	All-cause and cardiovascular mortality, stroke, hospitalization for heart failure, coronary revascularisation and MACE (CVD, non-fatal MI, non-fatal stroke or hospitalization for heart failure)	Elevated TSH was associated with higher all-cause and cardiovascular mortality in subgroup with ischaemic heart disease (n=461, adjusted and unadjusted models), but not in group with valvular heart disease (n=104).
Müller et al. (137)	Euthyroid patients undergoing implantation of an ICD device	115	Prospective unicentre cohort study	Mean 3.3 years	Cardiovascular mortality, appropriate ICD therapy	TSH within its reference range was not associated with mortality or ICD therapy.
Yang et al. (138)	Patients receiving cardiac resynchronization therapy	1,316	Retrospective unicentre cohort study	Median 3.6 years	All-cause mortality, appropriate ICD therapy	Elevated TSH was not associated with all-cause mortality, but with increased risk for appropriate ICD therapy.
Evron et al. (139)	US veterans receiving thyroid hormone replacement therapy	705,307	Retrospective cohort study based on a data warehouse system	Median 4 years	Cardiovascular mortality	Risk for CVD was elevated in both cohorts with TSH <0.1 mIU/L and > 5.5 mIU/L. Dose-dependent increase toward the more extreme phenotypes of dysregulation.

Elevated TSH levels were identified in seven studies as risk factors for MACE as well. In sum, these studies comprised about 720 000 subjects. In the community-based Wickham survey elevated TSH concentration predicted a higher mortality of ischaemic heart disease and an elevated rate of fatal and nonfatal events (122). A study including subjects with systolic heart failure found TSH elevations to be associated with increased all-cause and cardiovascular mortality, but this association was lost after adjustment (127). Similar associations were observed in subjects with myocardial infarction and community-dwelling adults aged 30 years and older (130, 134). In subjects with heart failure elevated TSH concentration was associated with increased hazard ratio for a composite end point (131). Patients undergoing cardiac surgery with ischaemic heart disease demonstrated elevated TSH concentration to be associated with higher all-cause and cardiovascular mortality (136).

A small study in subjects with cardiomyopathy found TSH concentration within its reference range to be positively associated with risk for ventricular arrhythmia events in both adjusted and unadjusted models (133). The cardiovascular mortality was also associated to within-reference TSH in the PREVEND study covering community dwelling subjects younger than 72 years (39).

Additionally, several studies did not identify a relation between TSH concentration and major cardiovascular end points. These studies included in sum about 15 000 subjects. In the large community-based Rotterdam study, the TSH concentration did not predict the risk for SCD (36). Likewise, the Health in Men Study did not observe an association of TSH concentration or its quartiles to a composite end point in more than 3 000 elderly men (132). TSH concentration was not associated with all-cause or cardiovascular mortality in the Newcastle 85+ study as well. This study included more than 600 elderly subjects (129). Two studies including subjects undergoing device implantation did not find an association of TSH levels with all-cause mortality (137, 138), but one of these studies observed elevated TSH to predict appropriate ICD therapy (138).

**Figure 6:**
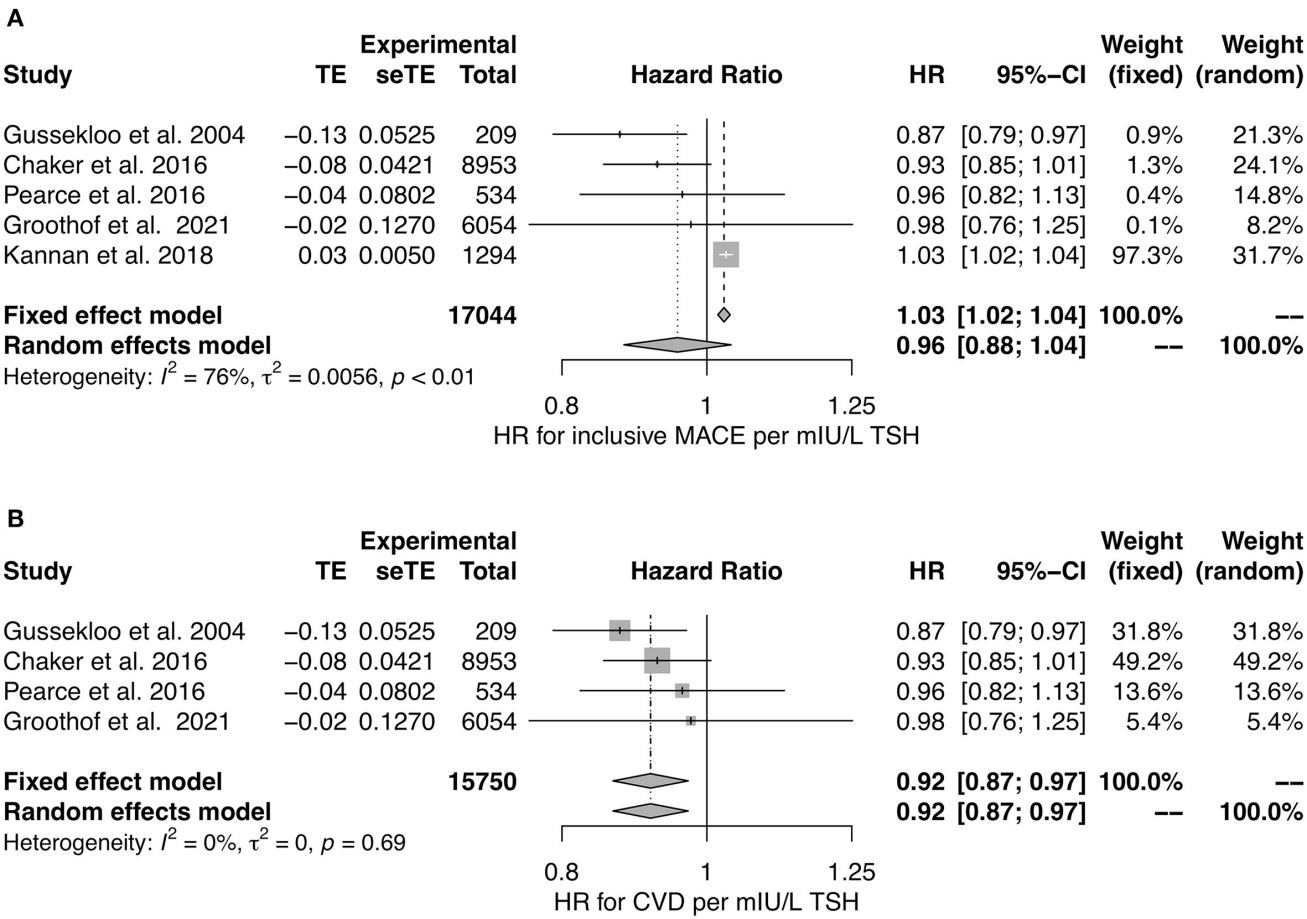
Hazard ratio for major cardiovascular events **(A)** and cardiovascular death **(B)** in relation to TSH concentration (36, 39, 120, 129, 131).

In a meta-analysis of studies reporting hazard ratios (36, 39, 120, 129, 131), we observed no association of TSH concentration to MACE in random effect models, but a significant negative association in both fixed and random effects models with CVD as end point (Figure 6). However, the number of eligible studies was small for CVD and the unexplained heterogeneity was substantial for MACE, as demonstrated by an *I*^2^ value of 75%.

U-shaped relationships between TSH and end points were described in 5 studies covering more than 720 000 subjects. In patients with heart failure both reduced and elevated TSH predicted increased mortality (125). Another study with patients receiving peritoneal dialysis observed both reduced and elevated TSH to be associated with increased mortality (128). This applied to unadjusted and adjusted models. After spline transformation, a study with community-dwelling individuals found a U-shaped association of TSH to total mortality (130). Similar observations were made in another population-based study that found low-normal, high-normal and elevated TSH to be associated with higher all-cause mortality than middle-normal TSH (135). This effect was partly mediated by cardiovascular disease. In the large population-based study by Selmer et al. both reduced and elevated TSH concentration was associated to cardiovascular endpoints (42). However, high TSH concentration predicted myocardial infarction only, whereas low TSH concentration was associated to MACE (CVD, non-fatal MI and non-fatal stroke), heart failure and all-cause mortality. An even larger data warehouse-based study by Evron et al. found a strong association of both reduced and elevated TSH concentration with cardiovascular mortality (139) in adults receiving thyroid hormone replacement therapy. In this study the increase in the adjusted hazard ratio was concentration-dependent, with a positive gradient toward both lower and higher TSH concentrations.

Elevated FT4 concentrations were associated with major endpoints in seven studies comprising in sum more than 720 000 subjects (Table 3). A positive association to the risk of SCD was observed in large community-based studies (36, 39, 120, 132). Two of these studies also observed FT4 concentration to be related to all-cause mortality (39, 120). The FT4 concentration predicted the hazard for a composite endpoint in subjects with heart failure (131). In a small study the FT4 concentration predicted the risk for appropriate ICD therapy and the hazard for ICD therapy-free survival (137). Two of the described studies also saw a positive association of within-reference FT4 concentration to major endpoints (36, 137). In a large data warehouse-based study including US veterans aged 18 or older on levothyroxine replacement therapy, both reduced and elevated FT4 concentrations were associated with the risk of cardiovascular mortality (139).

**Table 3:** Association between FT4 concentration and major cardiovascular outcome measures.

**Study**	**Study population**	**Number of included subjects**	**Study design**	**Evaluation period**	**Outcome**	**Main results**
Gussekloo et al. (120)	Population-based sample of elderly subjects aged 85 years or older	599	Population-based prospective cohort study	Mean 3.7 years	All-cause mortality, disability, depressive symptoms, cognitive function	Elevated FT4 concentration was associated with higher all-cause and cardiovascular mortality rate.
Chaker et al. (36)	Community-dwelling individuals included in the Rotterdam study	10,318	Population-based prospective cohort study	Median 9.1 years	Sudden cardiac death	FT4 concentration, even within its reference ranges was positively associated with hazard for sudden cardiac death. Risk for SCD was increased if FT4 was in the 3^rd^ tertile of the reference range unadjusted and adjusted models).
Pearce et al. (129)	Members of the Newcastle 85+ study, recruited from general (family) practices	643	Prospective cohort study	Up to 9 years	All-cause and cardiovascular mortality	FT4 concentration was not associated with all-cause or cardiovascular mortality after adjustment.
Kannan et al. (131)	Patients with heart failure enrolled in the Penn Heart Failure Study	1,365	Prospective multicentre cohort study	Median 4.2 years	Composite end point of all-cause mortality, cardiac transplant or VAD placement.	FT4 concentration was positively associated with hazard for composite end point.
Golledge et al. (132)	Community-recruited elderly men without known thyroid disease	3,712	Population-based prospective cohort study	Mean 9.5 years	Composite end point of cardiovascular death, myocardial infarction or stroke	Increased risk for the MACE and myocardial infarction in the highest quartile for FT4 concentration.
Groothof et al. (39)	community-dwelling individuals aged 28–75 years	6,054	Prospective cohort study	Mean 7.9 years	All-cause and cardiovascular mortality	FT4 concentration was positively associated with both all-cause and cardiovascular mortality (unadjusted and adjusted models).
Müller et al. (137)	Euthyroid patients undergoing implantation of an ICD device	115	Prospective unicentre cohort study	Mean 3.3 years	Cardiovascular mortality, appropriate ICD therapy	FT4 in the 2nd and 3rd tertiles of the reference range was positively associated with increased risk for appropriate ICD therapy. FT4 concentration associated to hazard for ICD therapy-free survival (unadjusted and adjusted models).
Evron et al. (139)	US veterans receiving thyroid hormone replacement therapy	705,307	Retrospective cohort study based on a data warehouse system	Median 4 years	Cardiovascular mortality	Risk for CVD was elevated in both cohorts with FT4 <9.0 pmol/L and > 24.5 pmol/L.

After adjustment, the Newcastle 85+ study did not find an association of FT4 concentration with all-cause or cardiovascular mortality. This study had a comparably small sample size of 643 elderly subjects (129).

**Figure 7:**
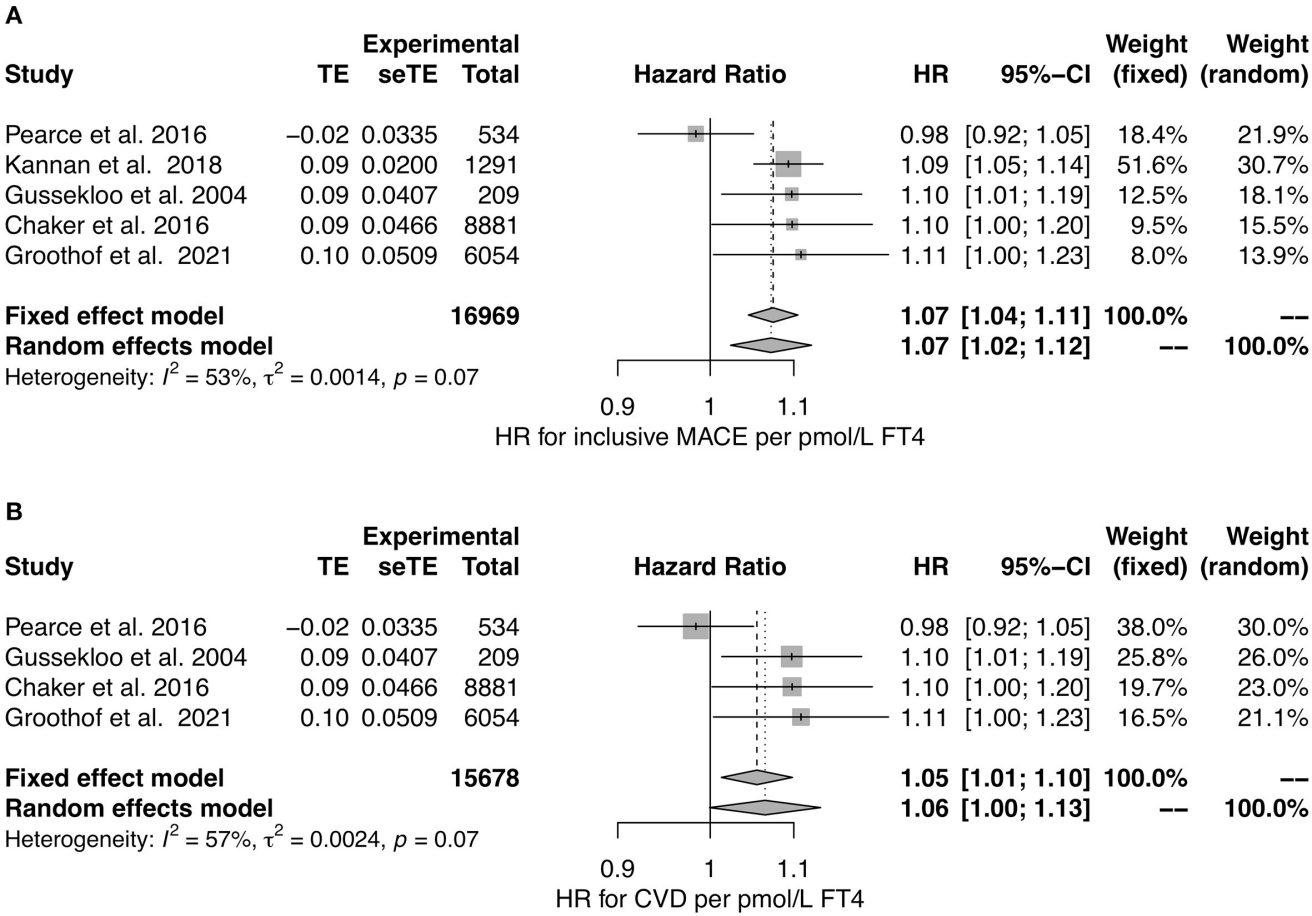
HR for MACE **(A)** and CVD **(B)** in relation to FT4 concentration (36, 39, 120, 129, 131).

Including studies reporting hazard ratios in a meta-analysis (36, 39, 120, 129, 131), we observed a significant association of FT4 concentration to MACE and a potential association to CVD (Figure 7). The heterogeneity between studies was moderate for both MACE and CVD with *I*^2^ values being 53 and 57%, respectively. Therefore, random effects models may be preferable over fixed-effects models to draw conclusions.

**Figure 8:**
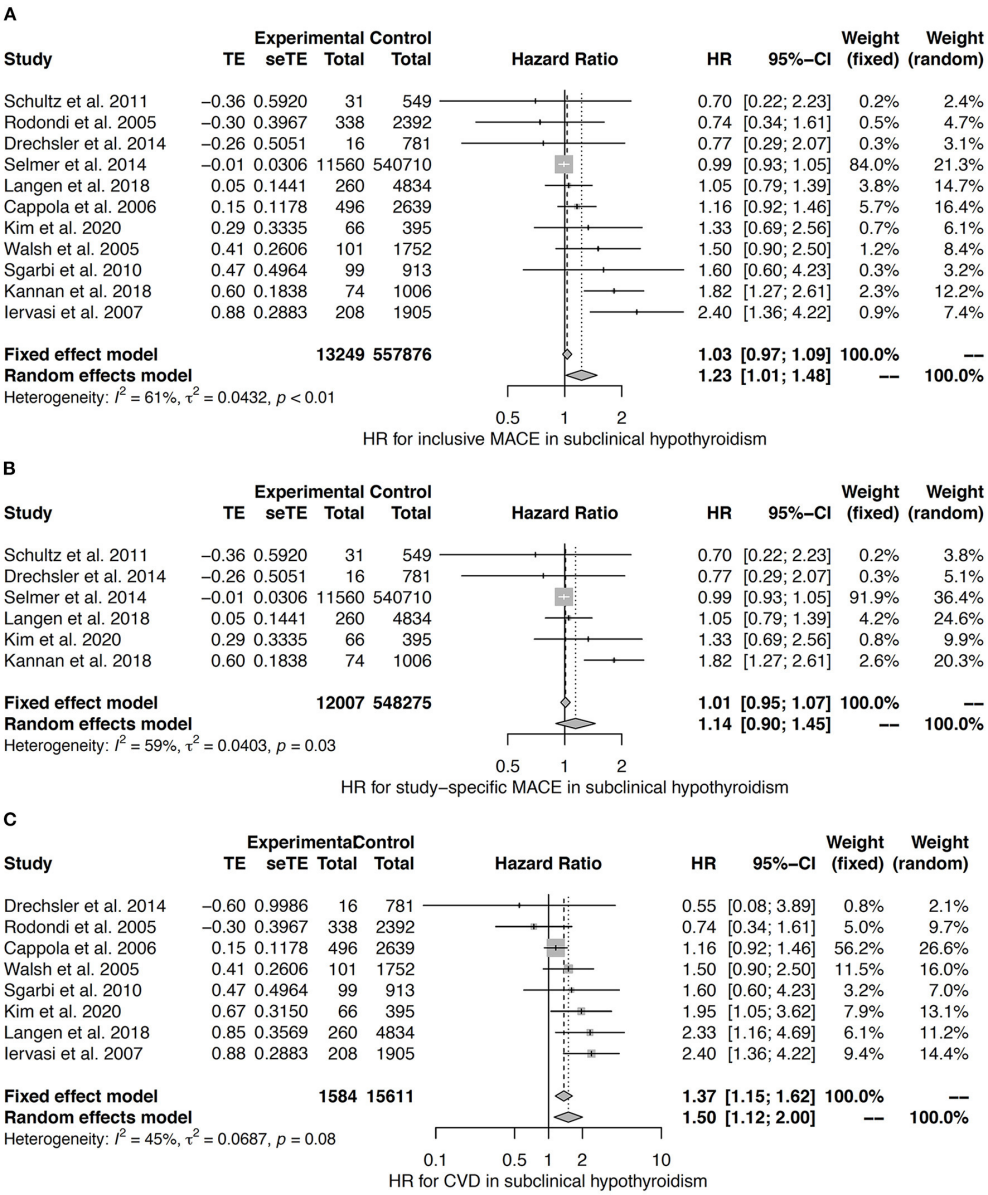
HR for inclusive MACE **(A)**, study-specific MACE **(B)**, and CVD **(C)** in subclinical hypothyroidism (42, 121, 123, 126, 130, 131, 136, 140–143).

The heterogeneity between studies was moderate to substantial in the meta-analysis for subclinical hypothyroidism (42, 121, 123, 126, 130, 131, 136, 140–143). Random effects models reveal an association to inclusive MACE, but not to study-specific MACE (Figures 8A,B). Both fixed effects and random effects models confirm subclinical hypothyroidism to predict CVD (Figure 8C).

**Figure 9:**
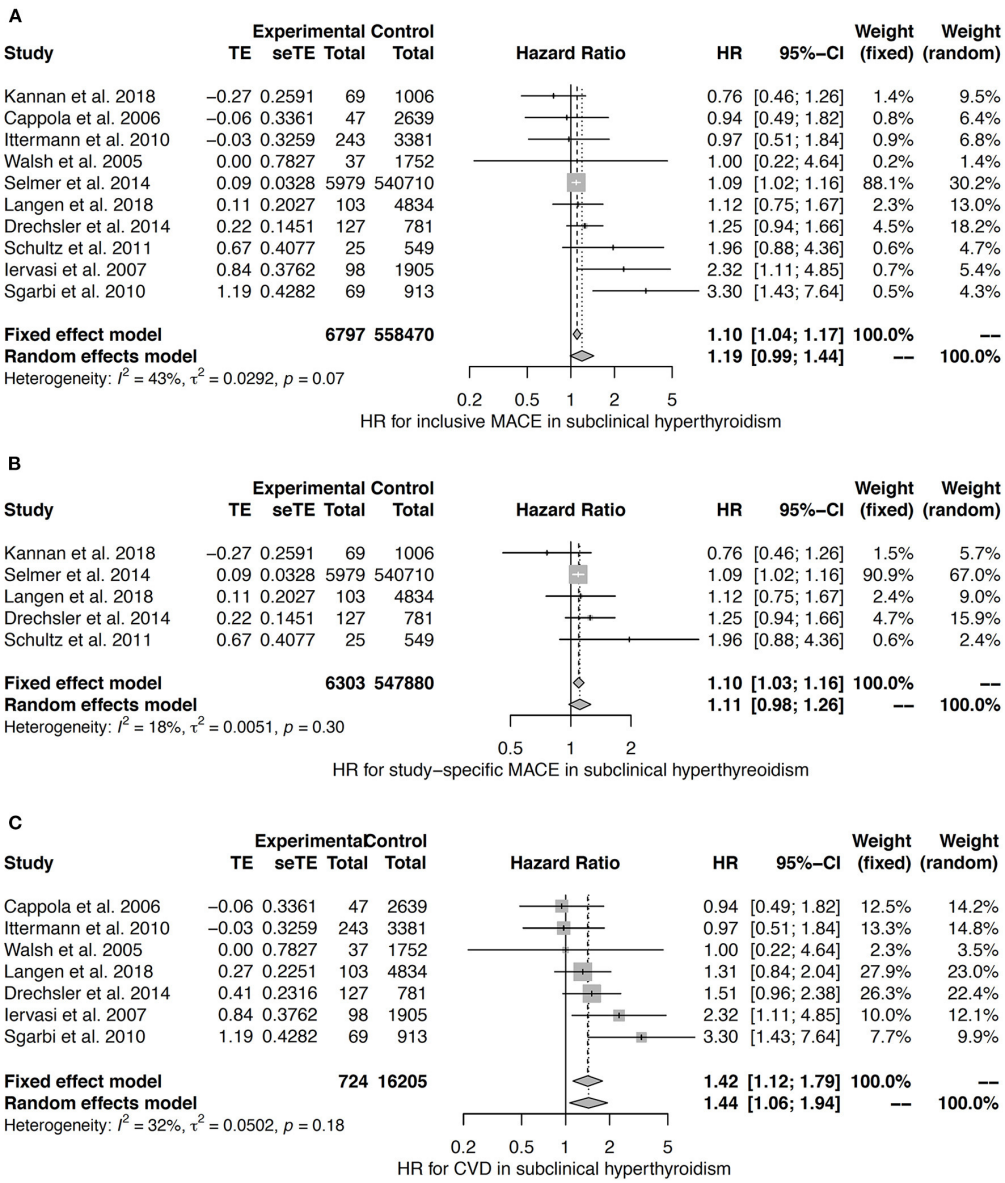
HR for MACE **(A,B)** and CVD **(C)** in subclinical hyperthyroidism (42, 121, 123, 126, 140, 142–144).

In the meta-analyses for subclinical hyperthyroidism the heterogeneity was very low (study-specific MACE) or moderate (MACE and CVD) (42, 121, 123, 126, 140, 142–144). The fixed effects model suggests a small effect with a hazard ratio of 1.10 with respect to both inclusive MACE and study-specific MACE (Figures 9A,B). An association to CVD was confirmed by both fixed effects and random effects models (Figure 9C).

The studies included in meta-analyses had a mean Newcastle-Ottawa score of 7.7, suggesting a sufficient quality of the available evidence. Funnel plots did not signify considerable bias, but support the assumption of heterogeneity among included studies. Detailed information is provided in the Supplementary material.

## Discussion

Both the generation of normal cardiac automaticity and the pathogenesis of arrhythmia are critically dependent on thyroid hormone signaling. Basic research identified multiple mechanisms linking thyroid hormone action to the formation of the normal and pathological heart rhythm. On a molecular level, classical thyroid hormone signaling (type 1 action) modulates the expression of multiple genes involved in the generation and conduction of excitation. On the level of electrophysiological processes, critical components of the rhythm-generating loops are sensitive to active iodothyronines. Interestingly, TSH has fundamentally opposing effects on generator loops, probably mediated *via* IP3 signaling.

Non-canonical thyroid hormone signaling has been linked to heart rate as well. The detailed mechanisms are not well understood up to now.

Although thyroid hormones increase the heart rate, they slow down the velocity of impulse conduction (θ), mainly due to down-regulation of critical components of gap junctions. Together with a shortened effective refractory period (ERP), this may lead to re-entrant tachycardia, if the wavelength of excitation


$$\begin{array}{l} \lambda =ERP\;\cdot \theta \end{array}$$

gets shorter than the length of potential anatomical re-entry circles. Necessary for this mechanism to come into effect is some heterogeneity in the myocardial tissue, e.g., due to the existence of scars forming the center of latent re-entry circles and to some electrophysiological variety of conducting tissue. This may explain why the probability of thyrogenic arrhythmia increases with age and in subjects with pre-existing cardiac disease.

From a clinical perspective, a relation of thyroid function to mortality is well established. Both the described physiological mechanisms and several prospective studies suggest this association to be mediated by arrhythmia.

With our search strategy we identified 32 publications investigating a potential relation between minimal deviations of thyroid function and major cardiovascular end points. Several studies with large sample sizes found reduced and/or elevated TSH concentration to predict cardiovascular or all-cause mortality, or composite MACE endpoints. Two studies described even a positive correlation of the TSH concentration within its reference range with cardiovascular events. However, several studies, even with large sample size, did not find an association of TSH concentration to major outcome measures. Our meta-analyses identified a negative relation of TSH to hazard ratios of CVD, but no clear association to MACE.

The situation was clearer for FT4 concentration. Six studies with a large cumulative sample size found a significant positive association between FT4 and cardiovascular events, including composite endpoints, mortality and appropriate ICD therapy. In two studies this association was also present if FT4 concentration was restricted to its reference interval. Only one study described both reduced and elevated FT4 concentrations to be associated to CVD (139), but this study included subjects on levothyroxine replacement therapy and may, therefore, represent slightly different pathophysiological mechanisms. *Via* meta-analysis we found a positive association of FT4 to MACE and a tendency for CVD.

The dissimilarity between these results, with a strong monotone prediction model for FT4, but a much less clear association of TSH to major endpoints, may be traced back to several causes. One may be the considerable heterogeneity of the results in the included studies, so that less powerful random effects models had to be preferred to fixed effects models, especially for the association of TSH concentration to MACE. Another explanation may be a U-shaped relation between TSH concentration and the risk for cardiovascular events (145). This form of nonlinear interaction was revealed by four studies that found both reduced and elevated TSH to predict mortality. Studies with negative results may have missed this complex association due to the use of over-simplified statistical models. It is corroborated, however, by our meta-analyses revealing both subclinical hypothyroidism and hyperthyroidism to predict cardiovascular mortality (Figures 8C, 9C).

**Figure 10:**
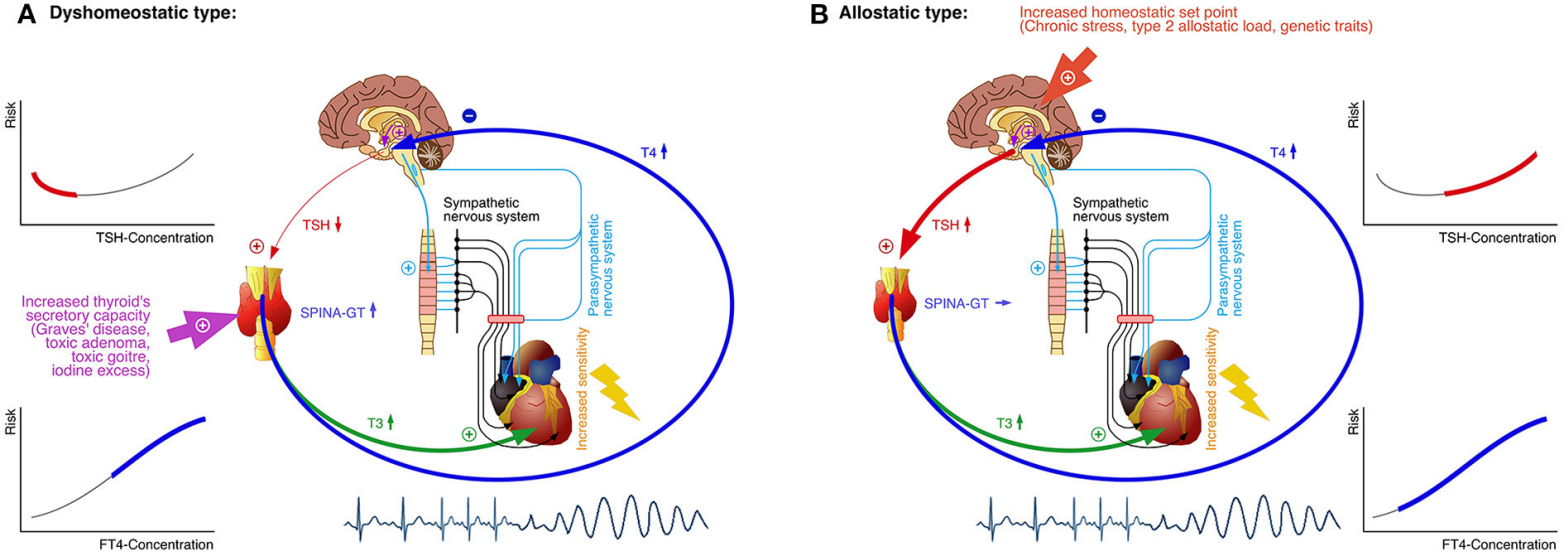
An integrated model for the association between thyroid homeostasis and major cardiovascular events. In the dyshomeostatic type of thyrogenic arrhythmia elevated FT4 concentration, caused by primary thyrotoxicosis, increases the risk for severe arrhythmia as a major cause for cardiovascular mortality. The TSH concentration is reduced in this case, represented by the left, declining, branch of the U-shaped relation between TSH level and risk **(A)**. In the allostatic type, mainly caused by type 2 allostatic load and genetic traits, the set point of thyroid homeostasis is raised, resulting in increased TSH and FT4 concentration and subsequently elevated risk for arrhythmia. This situation is mirrored in the right, rising, branch of the relation between TSH concentration and cardiovascular risk **(B)**. In any case, elevated concentrations of T3, T4 and other T3-agonistic thyroid hormones increase the sensitivity to catecholamines and sympathetic signaling (*via* upregulated expression of beta1 and beta2 adrenoceptors), thereby contributing to reduced stress tolerance. SPINA-GT, “gain of thyroid,” i.e., thyroid's secretory capacity (150).

Our results are consistent with the outcomes of previous studies and meta-analyses (146). However, a U-shaped link between TSH and cardiovascular risk is not straightforward to be explained from a physiological perspective. A recent study described very similar results for the link between thyroid function to Takotsubo syndrome, and the pathogenesis was explained by nosological heterogeneity, where two different scenarios overlapped (147). These considerations may be extended to cardiovascular endpoints in general (Figure 10). A dyshomeostatic type of thyrogenic arrhythmia may result from primary hyperthyroidism and other forms of non-central thyrotoxicosis. Here, the TSH secretion responds inversely to rising FT4 concentrations, so that FT4 is positively, but TSH negatively associated to the event rate (53, 148, 149). In an allostatic type of thyro-cardiac linkage, however, TSH concentrations rise with acute stress levels and long-term type 2 allostatic load, with consecutive, centrally-mediated, increasing T4 secretion (47, 56, 147). This case reflects a raised set-point of thyroid homeostasis. Therefore, both TSH and FT4 are positively associated to mortality and other major endpoints. Taken together, the blending of both types in population-based studies yields a U-shaped association between TSH concentration and the hazard of cardiovascular events, but a simpler monotonic prediction model for FT4 (Figure 10).

Multiple studies, reviews and meta-analyses described reduced T3 concentrations and other phenotypes of non-thyroidal illness syndrome (representing a type 1 allostatic response being also known as euthyroid sick syndrome or TACITUS—thyroid allostasis in critical illness, tumors, uraemia and starvation) to be linked to cardiovascular and general mortality and arrhythmia as well (56, 78, 151–153). Up to now, it is still not established whether low-T3 syndrome represents an adaptive response, a maladaptive mechanism or a combination of both (79). We did not address this topic in detail here and even excluded studies on TACITUS from the systematic review part, since this association may reflect common causes of mortality rather than a direct effect of low-T3 syndrome on cardiac electrophysiology. However, some non-classical thyroid hormones, especially 3,5-T2, which is thyromimetic and upregulated in critical illness, may increase both heart rate and risk for arrhythmia (152, 154).

Type 1 allostatic response in heart failure may to a certain extent limit our conclusions since FT4 concentrations could rise if the pathway *via* deiodinases to T3 formation is down-regulated (56). Decreased deiodinase activity may result from critical illness, but also from therapy with certain drugs, including amiodarone, beta-blockers and glucocorticoids (155). Additionally, the free fraction of thyroid hormones may increase due to drug interference with plasma protein binding, which may originate, e.g., from the use of furosemide, aspirin, heparin or other substances (156–158). Therefore, elevated FT4 concentration might as well partly reflect therapeutic decisions in subjects with cardiac failure. It is, however, unlikely that major bias arises from this fact since most of the described effects are rapidly settled by the hypothalamus-pituitary-thyroid feedback loop and, therefore, of temporary nature, whereas the follow-up time in most of the included studies extended to several years (55, 159).

The selection criteria for the systematic review may have led to the under-representation of studies reporting the risk in alternative form, e.g., in form of quantiles. This potential bias may have been mitigated by the fact that we included publications from other sources, e. g., own archives. Unfortunately, it was impossible to sensibly combine quantile-based studies in a meta-analysis, since the numbers of quantiles (tertiles, quartiles and quintiles were used) differed among the publications. Qualitative analysis suggests, however, an association of FT4 in the highest tertiles or quartiles to cardiovascular risk.

In summary, basic research and physiological studies suggest a strong link of thyroid hormones to cardiac rhythm generation and the pathogenesis of arrhythmia. The clinical evidence suggests a monotonic and unambiguous relation of FT4 concentration to arrhythmia, which seems to be one of the most important mediators of major cardiovascular endpoints. The relationship of TSH levels to arrhythmia, mortality and other outcome measures is less clear and best explained by a U-shaped link, which may reflect the overlap of two different scenarios, a dyshomeostatic type of thyrogenic arrhythmia resulting from ensuing primary thyrotoxicosis, and an allostatic response with an increased set point of the feedback loop. This hypothesis may serve as a starting point for future targeted research projects.

## Conclusions and outlook

Variations of thyroid function, even the slightest forms within the respective reference ranges of TSH and thyroid hormones, are strong predictors of major cardiovascular endpoints. However, the nature of this relation is more complex than previously assumed.

In addition to the well-established effects of primary thyrotoxicosis, central mechanisms of the hypothalamus-pituitary-thyroid (HPT) axis may play an important, but previously under recognized, role as well. This class of diseases includes secondary and tertiary hyperthyroidism in pituitary-related and hypothalamic disorders and in central resistance to thyroid hormone (RTH). These conditions are rare diseases, however, with respective prevalence of about 1 in 100 000 subjects. Much more common are adaptive responses representing type 2 allostatic load (160). Examples include post-traumatic stress disorder (PTSD), certain psychiatric diseases and long-term effects of social disparity. It could be demonstrated that conditions associated to type 2 allostatic load involve an elevated set point of the HPT axis and that they convey an increased risk for major cardiovascular events (5, 47, 56, 147, 161–163). The heterogeneity of pathophysiological mechanisms provides a plausible explanation for the U-shaped relation between TSH concentration and cardiovascular risk.

The new understanding of the dual etiology of the thyro-cardiac link has important therapeutic implications. First of all, the threshold for the treatment of thyrotoxicosis may be in future adjusted to include subclinical hyperthyroidism (SH) as well. This decision may be supported by results of a meta-analysis revealing a 24% increased risk of overall mortality in SH (164). However, the potentially beneficial effects of a more intensive correction of low-grade thyrotoxicosis have to be balanced against the risks resulting from treatment with thyrostatic agents and from definitive therapy (surgery or radioiodine treatment). The European Thyroid Association recommended treatment of SH in subjects of 65 years or older and in younger persons with concomitant cardiovascular disease (165).

The situation is more straightforward regarding the treatment of hypothyroidism. Here, it may prove advantageous if dosage titration algorithms for levothyroxine approach the target from below and prevent the free T4 concentration from entering the zone of the highest quartile of its reference range (166). This consideration also applies to the secondary prophylaxis of differentiated thyroid cancer (DTC). Accordingly, recent guidelines have also addressed cardiovascular concerns when overturning the previous recommendation of universal TSH suppression in low- and intermediate-risk DTC (167).

Treatment of type 2 allostatic load with a subsequently elevated set point of the HPT axis should address its origins. Sensible starting points include psychosocial support and lifestyle interventions (168–172). Early addressing of socioeconomic disparity and environmental factors may provide the basis for primary prevention of cardiovascular mortality (173, 174), and this may in part be due to a readjustment of thyroid homeostasis toward a healthier phenotype.

Where possible, future studies on the association of thyroid function with major cardiovascular endpoints should be based on validated criteria, including standardized MACE sets and uniform quantile definitions, in order to support replication, comparison and aggregation of results (118).

## Author contributions

PM, JD, and ML defined the selection criteria and screened all found studies for eligibility by abstract screening and full-text reviewing. JD maintained the study table and performed the meta-analyses. JD and PM drafted a first version of the manuscript. ML edited the text and contributed additional ideas, material, and text passages. All authors contributed to the article and approved the submitted version.

## Funding

Publication fees were defrayed *via* the Open Access Publication Funds of the Ruhr-Universität Bochum.

## Conflict of interest

The authors declare that the research was conducted in the absence of any commercial or financial relationships that could be construed as a potential conflict of interest.

## Publisher's note

All claims expressed in this article are solely those of the authors and do not necessarily represent those of their affiliated organizations, or those of the publisher, the editors and the reviewers. Any product that may be evaluated in this article, or claim that may be made by its manufacturer, is not guaranteed or endorsed by the publisher.
