# Supplementary material for: Minor perturbations of thyroid homeostasis and major cardiovascular endpoints—Physiological mechanisms and clinical evidence
Source: Front Cardiovasc Med. 2022 Aug 15;9:942971. doi: 10.3389/fcvm.2022.942971 (PMC9420854; doi:10.3389/fcvm.2022.942971)

Supplementary material accompanying the publication "Minor Perturbations of Thyroid Homeostasis and Major Cardiovascular Endpoints – Physiological Mechanisms and Clinical Evidence"

Patrick Müller, Melvin Khee-Shing Leow, Johannes W. Dietrich*

*** Correspondence:** Corresponding Author: johannes.dietrich@ruhr-uni-bochum.de

Supplementary table 1: Newcastle-Ottawa scores of included studies

Author	Year	S1	S2	S3	S4	C1	O1	O2	O3	NOS
Cappola et al.	2006	1	1	1	1	1	1	1	1	8
Chaker et al.	2016	1	1	1	1	1	1	1	1	8
Drechsler et al.	2014	1	1	1	1	1	1	1	1	8
Groothof et al.	2021	1	1	1	1	1	1	1	1	8
Gussekloo et al.	2004	1	1	1	1	1	1	1	1	8
Iervasi et al.	2007	1	1	1	1	1	1	1	1	8
Ittermann et al.	2010	1	1	1	1	1	1	1	1	8
Kannan et al.	2018	1	1	1	1	1	1	1	1	8
Kim et al.	2020	0	1	1	1	1	1	1	1	7
Langén et al.	2018	1	1	1	1	1	1	1	1	8
Müller et al.	2020	0	1	1	1	1	1	1	1	7
Pearce et al.	2016	1	1	1	1	1	1	1	1	8
Rodondi et al.	2005	1	1	1	1	1	1	1	1	8
Schultz et al.	2011	1	1	1	1	1	1	1	1	8
Selmer et al.	2014	1	1	1	1	0	1	1	1	7
Sgarbi et al.	2010	1	1	1	1	1	1	1	1	8
Walsh et al.	2005	1	1	1	1	1	1	1	1	8
Yang et al.	2020	0	1	1	1	1	1	0	0	5
		Case definition adequate? Representativeness of exposed cohort?	Representativeness of cases? Selection of non-exposed cohort?	Selection of controls Ascertainmant of exposure	Definition of controls Demonstration that outcome not present at start	Comparability of cases and control	Assessment of outcome	Follow-up long enough?	Adequacy of follow-up?	

Supplementary figure 1: Numbers of studies included in the meta-analyses with respect to different end-point criteria. Inclusive MACE: CVD or other major endpoints.

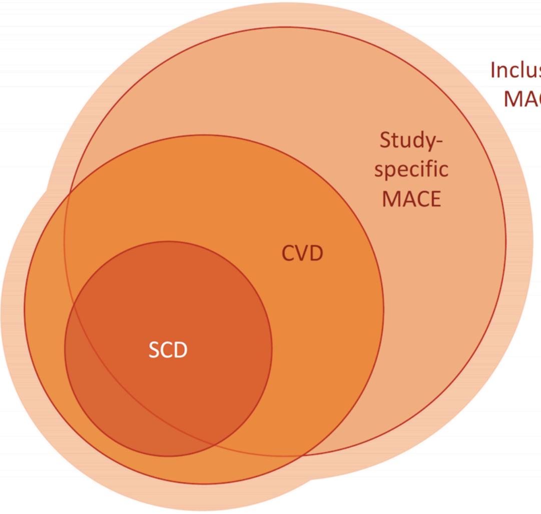

End point class	TSH	FT4	Subclinical hypo-thyroidism	Subclinical hyper-thyroidism
Inclusive MACE	5	5	11	10
Study-specific MACE	1	1	6	5
CVD	4	4	8	7
SCD	1	1	0	0

Supplementary figure 2: Funnel plots of meta-analyses with five or more studies

A: Inclusive MACE ~ TSH concentration:

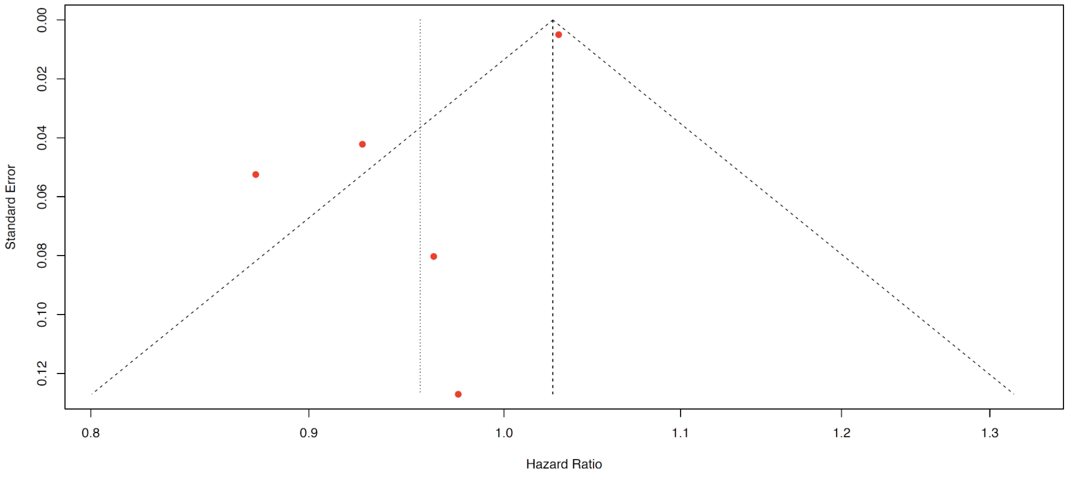

B: Inclusive MACE ~ FT4 concentration:

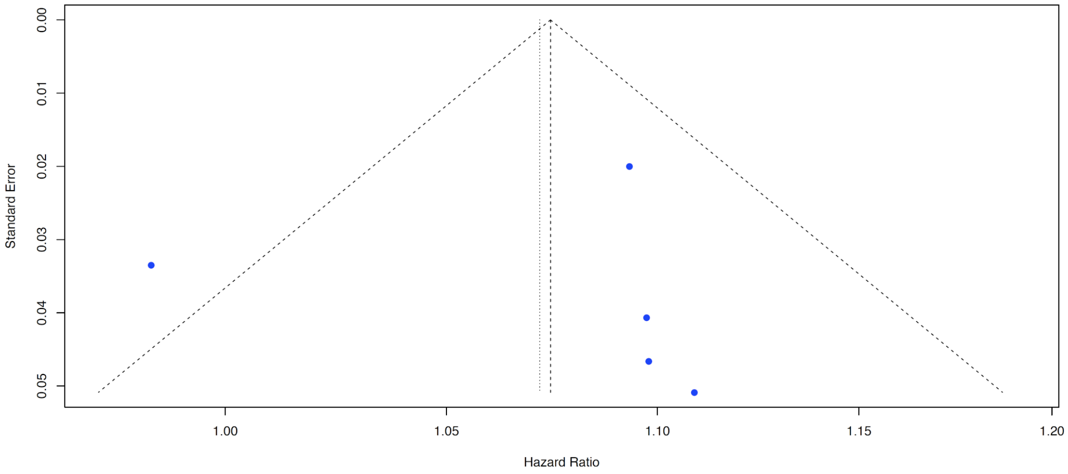

C: Inclusive MACE ~ Subclinical hypothyroidism:

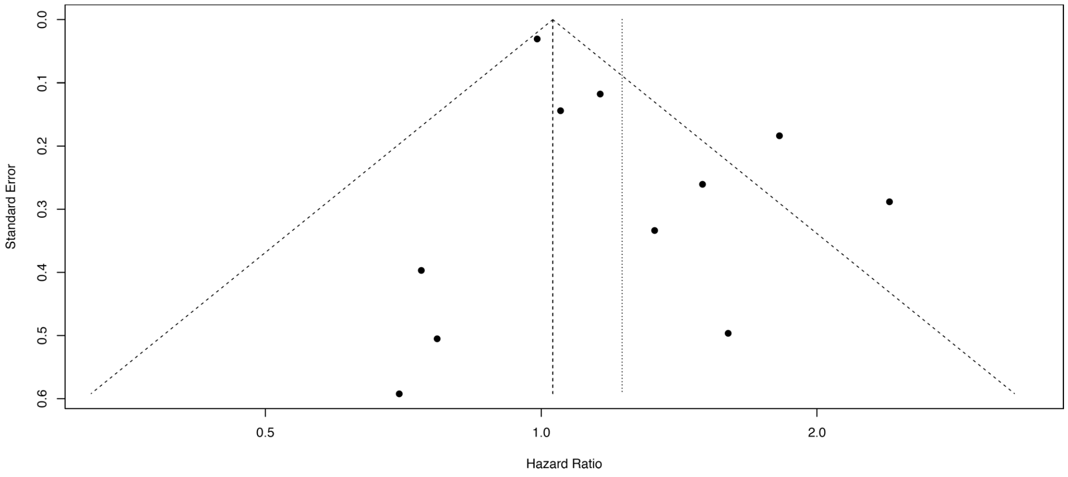

D: Study-specific MACE ~ Subclinical hypothyroidism:

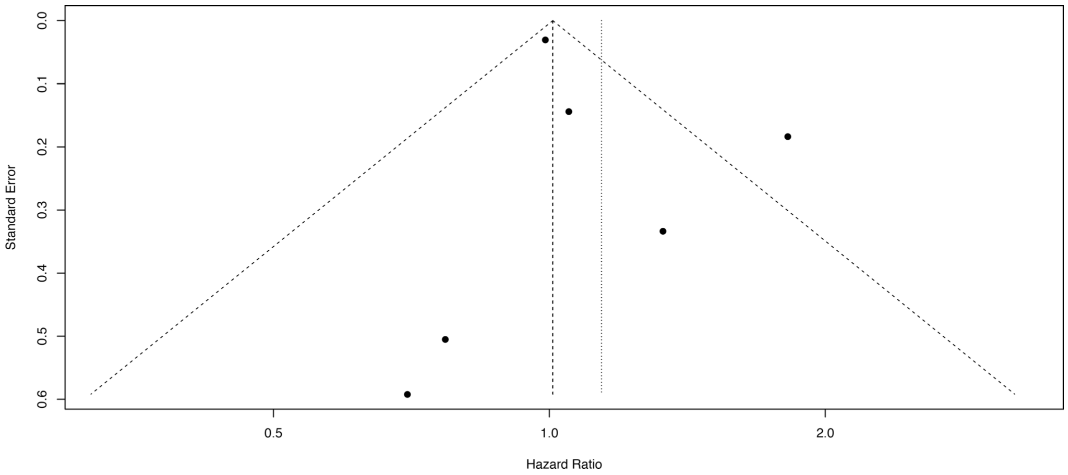

E: CVD ~ Subclinical hypothyroidism:

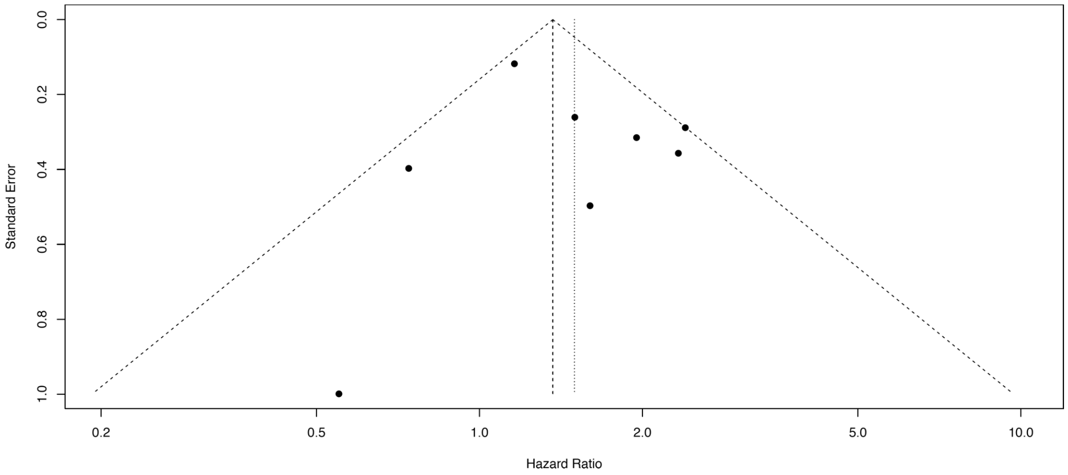

C: Inclusive MACE ~ Subclinical hyperthyroidism:

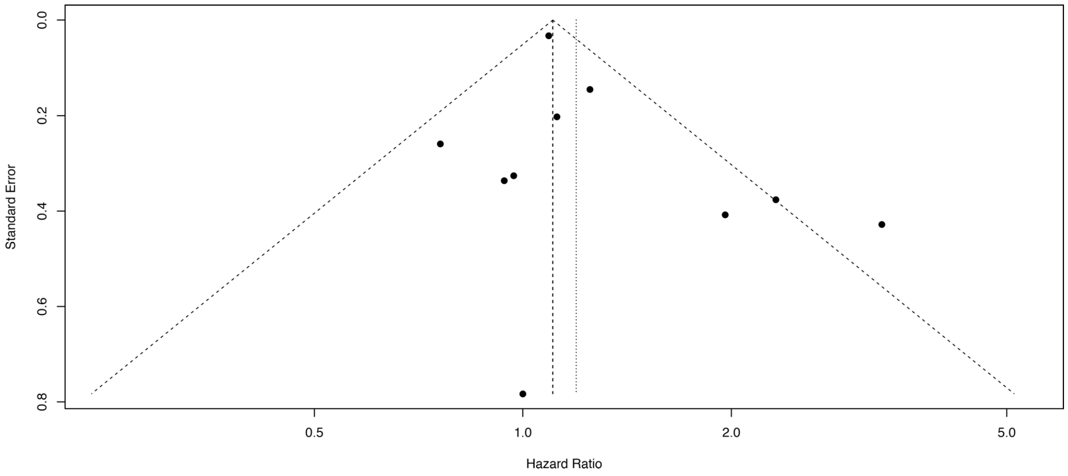

D: Study-specific MACE ~ Subclinical hyperthyroidism:

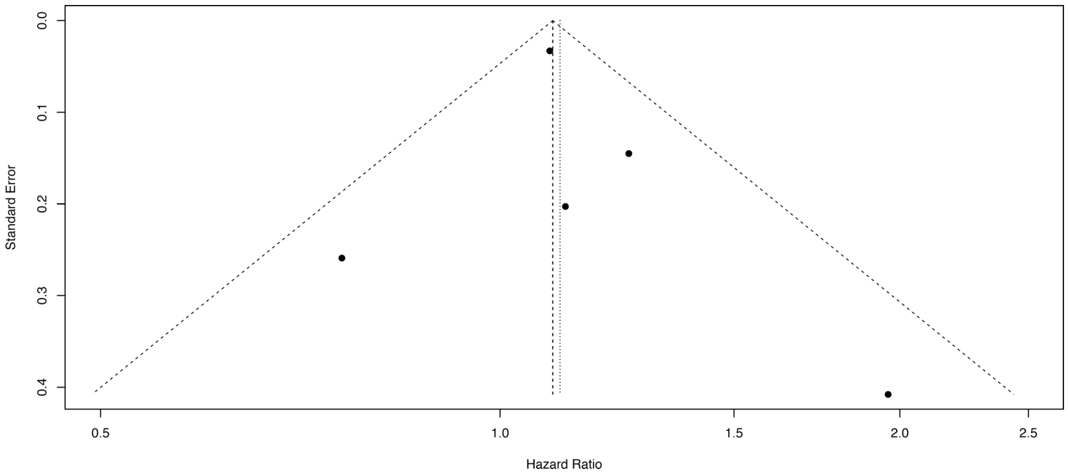

E: CVD ~ Subclinical hyperthyroidism:

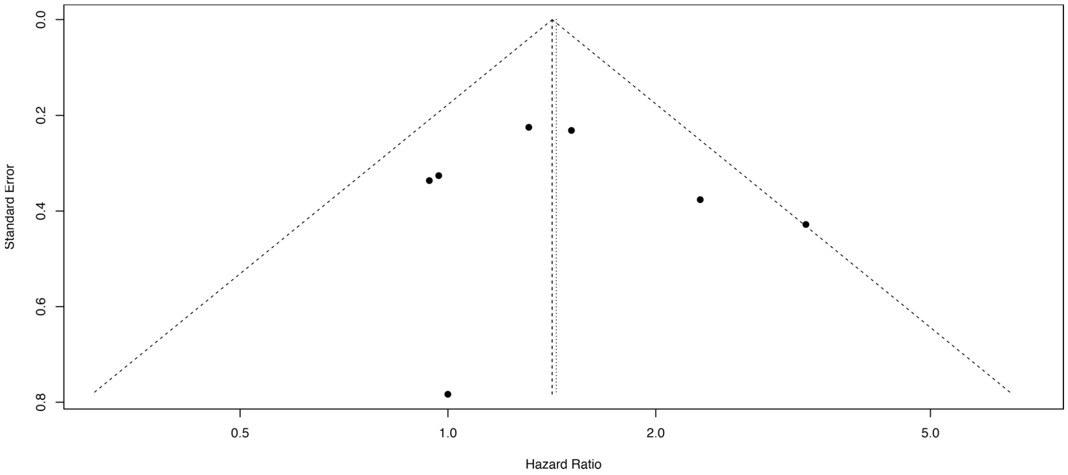

Supplement: Supplementary file 1 [file Data_Sheet_1.pdf]
